# New strategies for a sustainable ^99m^Tc supply to meet increasing medical demands: Promising solutions for current problems

**DOI:** 10.3389/fchem.2022.926258

**Published:** 2022-07-22

**Authors:** Mohamed F. Nawar, A. Türler

**Affiliations:** Department of Chemistry, Biochemistry, and Pharmaceutical Sciences (DCBP), Faculty of Science, University of Bern, Bern, Switzerland

**Keywords:** ^99m^Tc radiopharmaceuticals, ^99^Mo/^99m^Tc generators, nano-materials, photonuclear reaction, electrochemical separation, ^99^Mo supply chain, solvent extraction, column chromatography

## Abstract

The continuing rapid expansion of ^99m^Tc diagnostic agents always calls for scaling up ^99m^Tc production to cover increasing clinical demand. Nevertheless, ^99m^Tc availability depends mainly on the fission-produced ^99^Mo supply. This supply is seriously influenced during renewed emergency periods, such as the past ^99^Mo production crisis or the current COVID-19 pandemic. Consequently, these interruptions have promoted the need for ^99m^Tc production through alternative strategies capable of providing clinical-grade ^99m^Tc with high purity. In the light of this context, this review illustrates diverse production routes that either have commercially been used or new strategies that offer potential solutions to promote a rapid production growth of ^99m^Tc. These techniques have been selected, highlighted, and evaluated to imply their impact on developing ^99m^Tc production. Furthermore, their advantages and limitations, current situation, and long-term perspective were also discussed. It appears that, on the one hand, careful attention needs to be devoted to enhancing the ^99^Mo economy. It can be achieved by utilizing ^98^Mo neutron activation in commercial nuclear power reactors and using accelerator-based ^99^Mo production, especially the photonuclear transmutation strategy. On the other hand, more research efforts should be devoted to widening the utility of ^99^Mo/^99m^Tc generators, which incorporate nanomaterial-based sorbents and promote their development, validation, and full automization in the near future. These strategies are expected to play a vital role in providing sufficient clinical-grade ^99m^Tc, resulting in a reasonable cost per patient dose.

## 1 Introduction

Short-lived radionuclides continue to prove their crucial role in the development of nuclear medicine utilization in both diagnostic and therapeutic procedures. Effective therapy involves the delivery of a specific radiation dose to the tumor tissues either by the use of an external radiation source or internal radiotherapy techniques. For the former, hard gamma-ray emitters are favorably used, while a great need for the radionuclides with a low range of highly ionizing radiation emissions, such as alpha, beta and/or Auger electrons, are required to achieve internal therapy successfully (IAEA, 2009; Qaim, 2019). Definitely, a profitable therapy protocol strongly depends on the delivery of accurate information about the cancer lesion, such as the shape, size, and blood flow to the affected organ. To achieve this step, high-quality tumor imaging must be performed with the help of *in-vivo* diagnostic investigation tools. This method can be divided into two main categories, Positron Emission Tomography (PET), which includes the use of several positron emitters, and Single Photon Emission Computed Tomography (SPECT), in which radionuclides emitting low energetic gamma radiation are of great interest (Pimlott and Sutherland, 2011).

Particularly, SPECT radionuclides should have one or at least one main gamma energy line in the range of 100–200 keV. Table 1 displays the most commonly used radionuclides in SPECT imaging processes (Pimlott and Sutherland, 2011; Tàrkànyi et al., 2019).

**TABLE 1:** The predominantly used radionuclides in SPECT processes and their nuclear characteristics.

Radionuclide	Half-life	Mode of decay	Main photon energy, keV	Branching ratio (intensity), %	Availability	Remark
^99m^Tc	6.01 h	IT (99.996%)	140.51	89	^99^Mo → ^99m^Tc (^99^Mo/^99m^Tc generator)	
					^100^Mo(p,2n)^99m^Tc	
					^100^Mo(d,3n)^99m^Tc	
^123^I	13.27 h	EC, β^+^ (100%)	158.97	83	^122^Te(d,n)^123^I	• High cost
					^123^Te(p,n)^123^I	• Low availability
^111^In	2.80 d	EC (100%)	171.28	90	^112^Cd(p,2n)^111^In	• Relatively long half-life
			245.39	94	^111^Cd(p,n)^111^In	
^201^Tl	3.04 d	EC (100%)	167.43	10	^203^Tl(p,3n)^201^Pb → ^201^Tl	• Relatively long half-life
^67^Ga	3.26 d	EC (100%)	93.31	39.2	^68^Zn(p,2n)^67^Ga	• Relatively long half-life
			184.57	21.2	^67^Zn(p,n)^67^Ga	
			300.21	16.8	

The data were deduced from:

a https://www-nds.iaea.org/relnsd/vcharthtml/VChartHTML.html

b http://nucleardata.nuclear.lu.se/toi/

c https://www-nds.iaea.org/radionuclides/gamma_emitters.html

d https://www-nds.iaea.org/relnsd/vcharthtml/MEDVChart.html

To describe the pivotal role of ^99m^Tc, S. Esarey called it a vitally crucial diagnostic isotope used nearly once a second worldwide (IAEA, 2011). Undoubtedly, ^99m^Tc is the most extensively used diagnostic radionuclide in the SPECT family. It is currently involved in 80–85% of all *in-vivo* nuclear medicine diagnostic examinations (Boschi et al., 2017; Gumiela, 2018; Martini et al., 2018; Osso et al., 2012; Owen et al., 2014; Sakr et al., 2017). Annually, 30–40 million SPECT imaging studies have been conducted worldwide using ^99m^Tc solely (half of which in the United States). ^99m^Tc utilization is estimated to increase by 3–8% every year (Banerjee et al., 2000; Eckelman, 2009; Guerin et al., 2010; IAEA, 2013a; Chakravarty et al., 2013; Owen et al., 2014; Dilworth and Pascu, 2015; Gumiela, 2018; Haroon and Nichita, 2021). ^99m^Tc decays by a relatively short half-life (T_1/2_ = 6.01 h) and emits only one-gamma photon at low energy of 140.51 keV. This energy is ideally suited to penetrate the tissues and can be efficiently detected through SPECT cameras from outside the body, resulting in a high-quality image of the target organs with minimum radiation exposure dose to the patient. In addition, ^99m^Tc has unique coordination chemistry, which allows conjugation with a broad spectrum of diverse pharmaceutical molecules (IAEA, 2009; Dilworth and Pascu, 2015; Boschi et al., 2017; Gumiela, 2018). These ^99m^Tc-labelled compounds have been involved in the visualization of most organs (Vallabhajosula et al., 1989; Zolle, 2007; Leggett and Giussani, 2015; Araz et al., 2020; Urbano et al., 2020; Parihar et al., 2021). Moreover, the global on-demand availability of ^99m^Tc through the user-friendly ^99^Mo/^99m^Tc generators with reasonable cost-effectiveness has greatly encouraged nuclear medicine departments to conduct many research studies to explore its pivotal role in diagnostic imaging in detail (Mikaeili et al., 2018; Gan et al., 2020; Ávila-Sánchez et al., 2020). Table 2 highlights the wide-scale applicability of ^99m^Tc-based radiopharmaceuticals and provides the injection activity and the estimated exposure dose for each diagnostic procedure. Furthermore, based on its accessible availability and favorable nuclear properties, ^99m^Tc received considerable attention in multi-disciplinary fields. Recently, it has been involved in a variety of industrial radiotracer applications (Fauquex et al., 1983; Kantzas et al., 2000; Bandeira et al., 2002; Borroto et al., 2003; Berne and Thereska, 2004; Hughes et al., 2004; Thyn and Zitny, 2004; Dash et al., 2012; Pavlovicl et al., 2020).

**TABLE 2:** The most commonly used ^99m^Tc-based radiopharmaceuticals with their recommended injection activity and the estimated exposure dose.

Organ	^99m^Tc radiopharmaceuticals	Recommended adult injected activity, MBq^a^		Radiation dose estimation^b^	
		SNMMI	ENAM	Injected activity, MBq	Effective dose, mSv
Brain	^99m^Tc-ECD	555–1110	555–725	700	5.4
	^99m^Tc-HMPAO	555–1110	555–725	700	6.5
	^99m^Tc-MAA	40.7–151.7	60–78	78	0.9
Bone and bone marrow	^99m^Tc-MDP	740–1110	375–490	740	4.2
	^99m^Tc-HMPAO (WBC)	185–370	375–490	370	4.1
Heart	^99m^Tc-Pyrophosphate	370–555	N.A	370	2.1
	^99m^Tc-Tetrofosmin/sestaMIBI (exercise)	740–1480	900–1176 (One-day protocol)	740	5.2
			450–588 (2-day protocol min)		
			675–882 (2-day protocol max)		
	^99m^Tc-Tetrofosmin / sestaMIBI (resting)	740–1480	300–392 (One-day protocol)	740	5.6
			450–588 (2-day protocol min)		
			675–882 (2-day protocol max)		
	^99m^Tc-RBC	555–1110	600–784 (Blood pool)	555	3.9
	^99m^Tc-DMSA	74–222	73–95	90	0.8
	^99m^Tc-DTPA (IV)	37–1110	166–196 (normal renal function)	190	0.9
			150–196 (abnormal renal function)		
	^99m^Tc-MAG3	37–370	58–69	58	0.4
Liver	^99m^Tc-Sulfur/albumin colloid (IV)	148–222	N.A	185	2.6
	^99m^Tc-Sulfur/albumin colloid (oral)	18–74	N.A	46	1.0
	^99m^Tc-IDA	N.A	112–147	N.A	N.A
Lung	^99m^Tc-DTPA (Inhalation)	19.98–40.70	N.A	30	0.2
	^99m^Tc-Technegas	18.5–37	525–686	37	0.6
Spleen	^99m^Tc-RBCs	555–1110	30–39 (Denatured RBC)	555	3.9
Stomach	^99m^Tc-Pertechnetate	N.A	112–147	112	1.5
Thyroid	^99m^Tc-Pertechnetate	74–370	60–78	78	1.0
Parathyroid	^99m^Tc-MIBI	740–1480	N.A	N.A	N.A
Cancer cells identification	^99m^Tc-SestaMIBI	N.A	675–882	N.A	N.A

(ENAM), European Association of Nuclear Medicine; (SNMMI), the Society of Nuclear Medicine and Molecular Imaging.

a The activity doses were calculated for body weights from 50 to 68 Kg.

b The exposure doses are based on (ICRP, 2015).

The activity doses were taken from https://www.eanm.org/publications/dosage-calculator/ and http://www.snmmi.org/ClinicalPractice/doseTool.aspx (accessed 8.6.2022).

Despite the central role of ^99m^Tc for different diagnostic medical investigations as well as recent industrial implementations, its limited availability at the usage sites is a real difficulty. The primary objective of this review paper is to provide a detailed and comprehensive evaluation of the different production routes of ^99m^Tc that have commercially been exploited. In addition, new production strategies that could potentially promote efficient utilization of ^99m^Tc in the near future are highlighted. In the following sections, the paper discusses the different production routes of the parent ^99^Mo either by nuclear reactors or particle accelerators, the direct production technology of ^99m^Tc at a cyclotron, and the different ^99^Mo/^99m^Tc generator systems and their role in supplying ^99m^Tc. Furthermore, the paper underlines the advantages, outcomes, and technical challenges of the different ^99m^Tc production approaches along with their current situation and the possible future perspectives.

## 2 Production and supply of ^99^Mo

**FIGURE 1:**
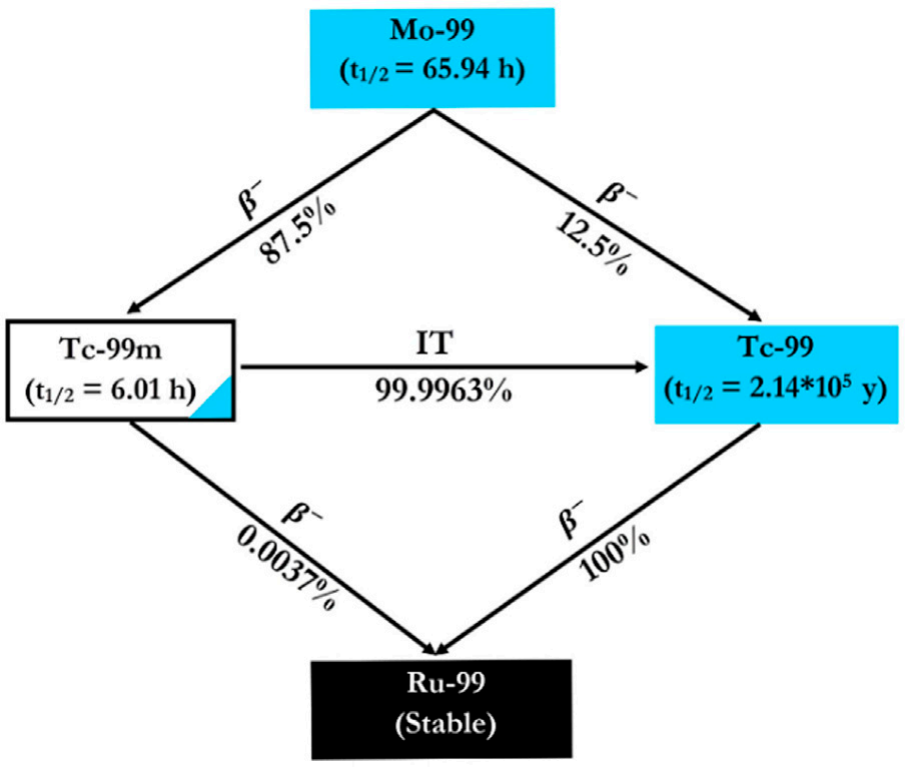
The decay scheme of ^99^Mo and ^99m^Tc (Hidalgo et al., 1967).

**FIGURE 2:**
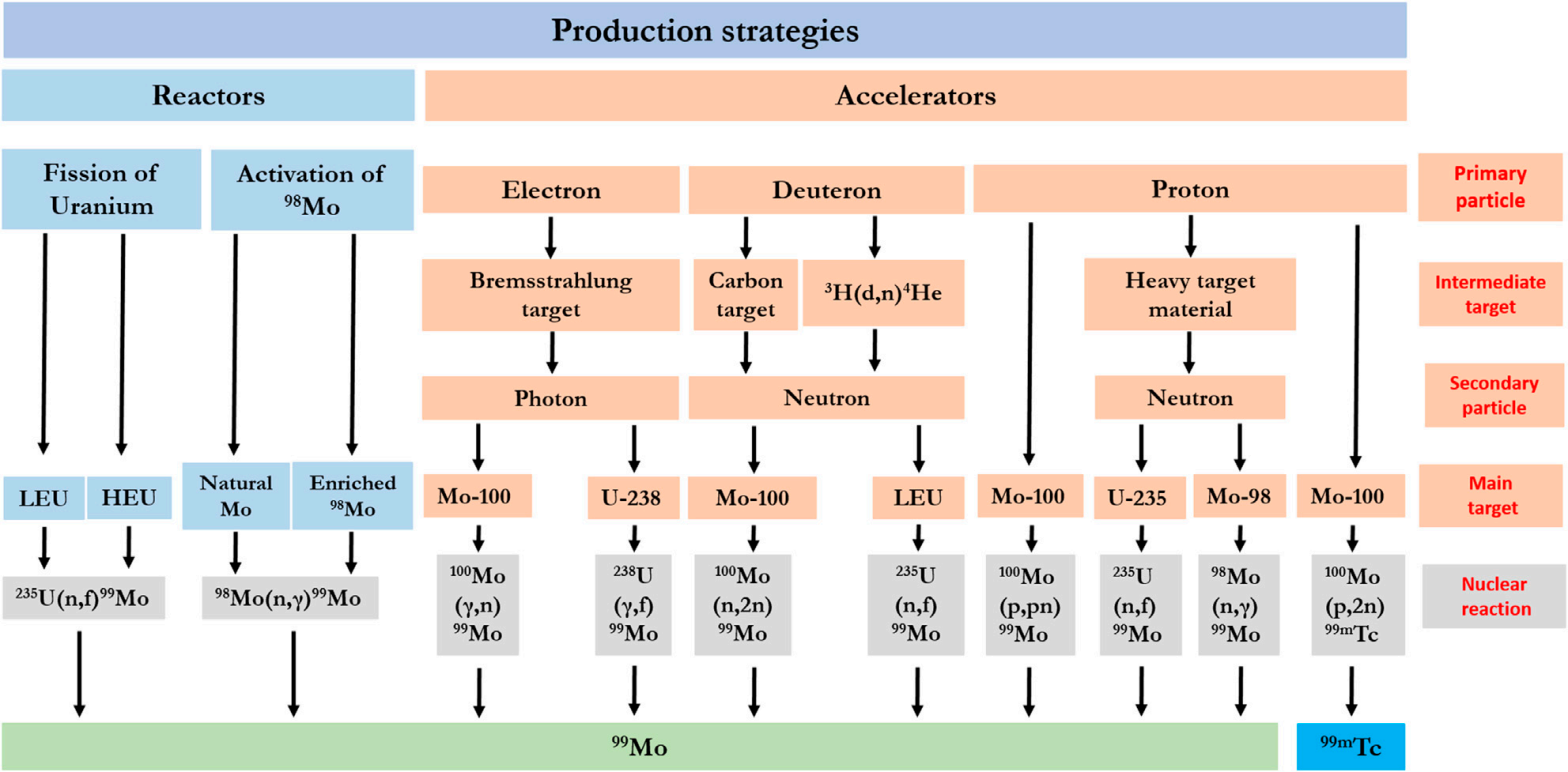
The potential production routes of ^99^Mo and ^99m^Tc. Abbreviations: HEU: Highly Enriched Uranium; LEU: Low Enriched Uranium.

The increasing interest in ^99m^Tc-labelled radiopharmaceuticals for diagnostic purposes has heightened the need for securing a sufficient supply of ^99^Mo. Practically, almost all of the used clinical-grade ^99m^Tc in nuclear medicine is derived from ^99^Mo/ ^99m^Tc generators. In this system, ^99^Mo (T_1/2_ = 65.94 h) yields ^99m^Tc by beta β^−^ decay, as shown in Figure 1 (Boyd, 1982a; Hidalgo et al., 1967; IAEA, 2017, 2013a; NEA, 2017, 2010). ^99^Mo is produced with the use of one of two main facilities; nuclear reactors or particle accelerators. Figure 2 displays the potential production routes of ^99^Mo and ^99m^Tc.

### 2.1 Reactor-based ^99^Mo production

The medical isotope ^99^Mo can be produced in nuclear reactors through two primary routes (IAEA, 1999):

#### 2.1.1 Fission-produced ^99^Mo

Undoubtedly, the neutron-induced uranium fission technique is widely considered the “gold standard” for the large-scale ^99^Mo supply for medical applications. Actually, over 95% of all ^99^Mo used for the production of medical-grade ^99m^Tc is available from the fission of uranium targets in nuclear reactors. These targets can be categorized according to the content of the fissile ^235^U radioisotope (IAEA, 1999, 2002, 2005; NAP, 2016):

1. Natural uranium: ^235^U content is about 0.72%.
2. Low Enriched Uranium (LEU): 0.72% < ^235^U content <20%.
3. Highly Enriched Uranium (HEU): 20% ≤ ^235^U content ≤90%.
4. Weapons-grade Enriched Uranium: ^235^U content ≥90%.

Generally, a neutron-induced fission reaction occurs when a target of a heavy fissionable element, such as ^235^U, is introduced into the reactor core. Hence, the ^235^U nucleus absorbs a thermal neutron and undergoes a fission reaction, resulting in two fission fragments. These comprise about 200 different radionuclides, including ^99^Mo, according to the reaction U235(n,f)M99o . The resulting ^99^Mo is called a Non-Carrier Added (NCA) product and possesses high specific activity defined as the ^99^Mo radioactivity per unit molybdenum mass. The fission yield for this reaction is about 6.132% (Haroon and Nichita, 2021; Grummitt and Wilkinson, 1946; Hyde, 1964; IAEA, 2013a, 2008, 2003). Other molybdenum isotopes, namely ^97^Mo, ^98^Mo, and ^100^Mo, are also produced with a total fission yield of 18.1%. These stable isotopes may dilute the ^99^Mo final activity. However, the specific activity of fission ^99^Mo is 1000 folds higher than the specific activity of ^99^Mo produced from neutron-activated ^98^Mo, as detailed in Section 2.1.2 below (Sameh and Ache, 1989). Table 3 shows a comparison between the production routes of ^99^Mo in a nuclear reactor.

**TABLE 3:** The evaluation of reactor-based ^99^Mo production technologies.

Criteria		Uranium fission		Neutron activation		
Target	Material	LEU	U-Al alloy	Natural ^98^Mo	MoO_3_	
			U-metallic foil		Mo metal	
		HEU	U-Al alloy	Enriched ^98^Mo	MoO_3_	
					Mo metal	
	Availability	Restricted to few producers		Broadly available		
Production process	Nuclear reaction	^235^U (n,f)^99^Mo		^98^Mo (n,γ)^99^Mo		
	Cross-section, barn	586 (× 6% fission yield)		Thermal flux	0.13	
				Epithermal flux	6.7±0.3	
	Yield	>10,000 Ci ^99^Mo/g Mo		Natural ^98^Mo target	∼0.2–1 Ci ^99^Mo/g Mo	Influenced by the neutron flux capacity
				Enriched ^98^Mo target	≥4 Ci ^99^Mo/g Mo	
	Specific activity	High specific activity product		Low specific activity product		
	Production facility	Limited to a small number of irradiation sites		More than 50 reactors with high neutron flux (>10^14^ n/cm^2^s) are geographically well-distributed		
	Co-production isotopes	200 different radioisotopes such as ^131^I and ^133^Xe		^99^Mo solely		
Chemical Processing	Separation step	Mandatory		Can be avoided		
	Feasibility	Complicated and involves hazardous materials		Simple and does not include any hazardous substances		
	Laboratories	Limited		Globally available		
Concerns	Proliferation risks	High		Negligible		
	Cost	High		Low		
	Waste	50 Ci waste per production of only 1 Ci ^99^Mo		Negligible		
Maturity	Status	Well-established technology		Growing technology		
	Capability	Covers >95% of the global demand		Used on small-scale production in some countries		
Licensing and approval	Nuclear regulatory	Approved		Approved		

##### 2.1.1.1 Global supply chain of fission-produced ^99^Mo

The availability of a sufficient ^99^Mo supply involves a sequence of connected steps with high complexity. These steps can be defined as the ^99^Mo supply chain. In the first step, uranium targets are fabricated and tested. Then, these targets are shipped to the irradiation facilities where the production of ^99^Mo takes place. After that, the irradiated targets are transported to well-equipped processing facilities comprising hot cells, where the separation and purification of ^99^Mo from other fission products are accomplished. Afterward, the purified carrier-free ^99^Mo solutions are eventually used for the ^99^Mo/^99m^Tc generator assembly process. Subsequently, these generators are globally distributed to provide ^99m^Tc of high quality, ready to use for different medical needs (NAP, 2018, 2016).

**FIGURE 3:**
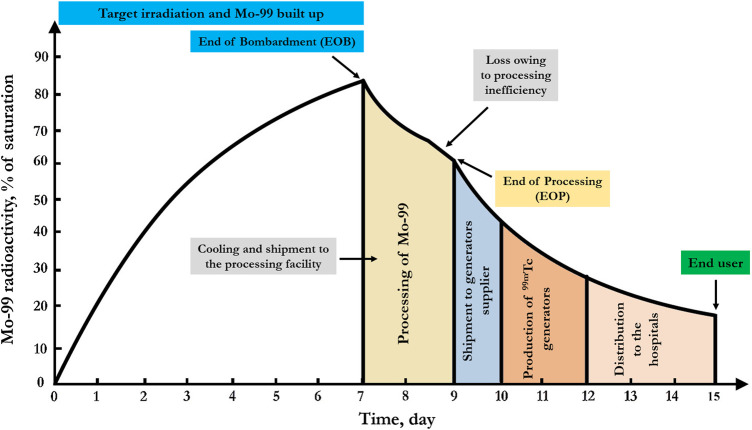
The schematic demonstration of the fission-produced ^99^Mo supply chain, including target irradiation, the production of ^99^Mo, and the 6-day Curie expression. The produced ^99^Mo activity from the supply chain is approximately 22% of the total irradiated activity in case of 100% recovery of ^99^Mo from the irradiated targets. In the same manner, if the processing efficiency is 90%, the expected remaining activity is about 17% of the total produced activity.

Based on the fact that ^99^Mo decays with a relatively short half-life (T_1/2_ = 65.94 h), all previous steps need to be achieved as rapidly as possible to minimize radioactive decay losses. The quantity of ^99^Mo produced is defined with the term “six-day Curie”. This term represents how much ^99^Mo radioactivity remains 6 days after the end of processing. Figure 3 highlights the six-day Curie concept through the ^99^Mo supply chain (Paterson et al., 2015). The “six-day Curie” concept has been introduced by the manufacturers as a base for calibrating the sales price. However, it is not very suitable to express the losses of ^99^Mo from the end of bombardment to arrival at the end user since neither transport to the processing facility, processing time, chemical yield, and transport times to the end user are accounted for.

Close to 10,000 uranium targets (among them 8,000 targets involving HEU) are consumed annually to afford a ^99^Mo supply for nuclear medicine utilization. There are, at present, only four agencies that can manufacture and supply targets to the irradiation facilities. These laboratories are operated under the authority of the Atomic Energy Commission of *Argentina* (CNEA), *Argentina*; Canadian Nuclear Laboratories (CNL), Canada; Company for the Study and Production of Atomic Fuels (CERCA), France; and Nuclear Technology Products Radioisotopes (NTP), South Africa (NAP, 2018, 2016).

The neutron bombardment of the targets can be carried out in research reactors of high neutron flux, which is usually in the range of ≥ 10^14^ n/cm^2^s. Table 4 illustrates the potential irradiation reactors and their production volume (Zhuikov, 2014; NAP, 2018; NEA, 2019). The ^99^Mo production capability of these reactors covers more than 95% of the global medical need for ^99^Mo. The target irradiation process typically lasts five to 7 days until the ^99^Mo growth reaches 80% of the saturation yield. At this point, the overall ^99^Mo activity remains unchanged. That is because the quantity of ^99^Mo produced by the target irradiation equals the loss of ^99^Mo activity due to the radioactive decay (Paterson et al., 2015; NEA, 2017).

**TABLE 4:** The producers of fission ^99^Mo and their global production volume.

Irradiation facility^a^			Country	Irradiated target	Neutron flux, n/cm^2^S	Year commissioned	Estimated available capacity, six-day Ci/week	Production of ^99^Mo, Week/year	Estimated available production capacity by 2024^b^, six-day Ci/year	Estimated global market share, %	Expected shutdown, year
Global producers	BR-2		Belgium	HEU/LEU	1.0E15	1961	6,500	21	136,500	17	2026
	HFR		Netherlands	HEU/LEU	2.7E14	1961	6,200	39	241,800	29	2026
	LVR-15		Czech Republic	HEU/LEU	1.5E14	1957	3,000	30	90,000	11	2028
	Maria		Poland	HEU	3.5E14	1974	2,200	36	79,200	9	2040
	OPAL		Australia	LEU	3.0E14	2006	2,150	43	92,450	11	2057
							+1,350^c^		+58,050^c^	7	
	Safari-1		South Africa	LEU	2.4E14	1965	3,000	44	130,700	16	2030
	NRU		Canada	HEU	4.0E14	1957	40% (Global market)	N.A	N.A	N.A	2018 Retired
	Osiris		France	HEU	1.7E14	1966	8% (Global market)	N.A	N.A	N.A	2015 Retired
Regional producers	RIAR	RBT-6	Russia	HEU	1.4E14	1975	540	50	27,000	Domestic use	2025
		RBT-10		HEU	1.5E14	1983					2025
	KAPROV	WWR-c		HEU	2.5E13	1959	350	48	16,800	Domestic use	2025
	RA-3		*Argentina*	HEU	4.8E13	1961	500	46	23,000	Domestic use	2027

The information is derived from:

The IAEA’s Research Reactor Database (RRDB): https://nucleus.iaea.org/rrdb/#/home (accessed 8.6.2022), NEA, 2019, Zhuikov, 2014, and NAP, 2018.

a BR-2, Belgian Reactor 2; HFR, high flux reactor; OPAL, open pool australian light water; NRU, national research universal; SAFARI-1, South African Fundamental Atomic Research Installation 1; RIAR, the Research Institute of Atomic Reactors; and N.A, Not Available.

b Available production capacity: is the upper limit of ^99^Mo production capability that can be achieved on a routine operating schedule.

c Additional ^99^Mo production capability owing to the engagement of a new processing facility, namely, the ANSTO, Nuclear Medicine (ANM) project, which started in May 2019.

After the irradiation step, the targets are left to cool down, allowing short-lived and ultra-short-lived fission products to decay. Then, the irradiated targets are moved to the processing facilities where the recovery of ^99^Mo from other fission products is conducted, and a pure ^99^Mo-molybdate solution is prepared. Currently, there exist only five large-scale processing centers that can perform this task. Table 5 shows the five main processing facilities and their global ^99^Mo supply (IAEA, 2013a, 2015; NAP, 2016,; NEA, 2019).

**TABLE 5:** The main uranium fission processing facilities and their ^99^Mo supply capacities.

Processing facility^a^		Country	Target	Chemical process^b^	Estimated available supply capacity, six-day Ci/week	Production of ^99^Mo, week/year	Estimated available supply capacity by 2024^c^, six-day Ci/year	Estimated global market share, %	Expected shutdown, year
Global producers	ANSTO	Australia	LEU	Alkaline	2,150	43	92,450	13	2057
	^d^ANM project				+1,350 ^**C**^		+58,050 ^**C**^	8	
	^e^Curium	Netherlands	LEU	Alkaline	5,000	52	260,000	37	N.A
	IRE	Belgium	HEU	Alkaline process	3,500	49	171,500	24	2028
	NTP	South Africa	LEU	Alkaline	3,000	44	130,700	18	2030
	^f^CNL/Nordion	Canada	HEU	Acidic	29% (Global market)	N.A	N.A	N.A	stopped
	Mallinckrodt	Netherlands	HEU	Alkaline	24% (Global market)	N.A	N.A	N.A	stopped
Regional producers	RIAR	Russia	HEU	Alkaline	540	50	27,000	Domestic use	2025
	KAPROV		HEU	Alkaline	350	48	16,800	Domestic use	2025
	CNEA	*Argentina*	LEU	Alkaline	500	46	23,000	Domestic use	2027

The information is derived from: 1 NEA, 2019, 2 NAP, 2018.

a ANSTO: australian nuclear science and technology organization; ANM: ANSTO, nuclear medicine; IRE: national institute for radioelements; NTP: nuclear technology products radioisotopes; RIAR: the Research Institute of Atomic Reactors; and N.A: Not Available.

b The alkaline process is fully compatible with the U-Al alloy targets and offers the advantage of ^131^I co-production capability.

c Available supply capacity: is the upper limit of ^99^Mo supply capability that can be achieved on a routine operation framework.

d Additional ^99^Mo supply capability owing to the engagement of a new processing facility, namely, ANSTO, Nuclear Medicine (ANM), which started in May 2019.

e Curium was established through a merger between Mallinckrodt Nuclear Medicine and Ion Beam Applications Molecular (IBAM). http://www.mallinckrodt.com/about/news-and-media/2197068 (accessed 8.6.2022).

f CNL/Nordion terminated their ^99^Mo processing activities at the end of 2016.

The uranium fission method deserves careful attention owing to its capability to provide adequate amounts of ^99^Mo in a carrier-free form with high specific activity, which can satisfy the global medical need. However, the application of this technology faces inherent critical challenges. For instance, proliferation concerns are dramatically growing owing to the use of massive amounts of HEU targets. Nearly 80% of the global demand of ^99^Mo is produced using uranium targets containing up to 93% of enriched ^235^U. During the production steps, about 50 kg of weapons-grade HEU are usually handled, and only a very minute amount (∼3%) is utilized in the entire process (NAP, 2009; NEA, 2010; IAEA, 2015). In order to eliminate these nuclear proliferation issues, HEU targets have to be fully substituted by LEU or natural uranium targets (Wu et al., 1995; Cols et al., 2000; Conner et al., 2000). In addition, the aging fleet of the currently used irradiation facilities is also of concern. Approximately 95% of the global supply of ^99^Mo depends on only six reactors. With the exclusion of OPAL, all the other five reactors have been in service for more than 50 years. Lately, a number of unplanned shutdowns have taken place, and consequently, the international medical community has severely suffered from a ^99^Mo shortage (Gumiela, 2018). In case of any unexpected future interruption, a renewed ^99^Mo supply crisis cannot be ruled out and could seriously affect many countries due to the expected rise in the cost of dose per patient (NEA, 2011).

Moreover, the separation and purification of ^99^Mo from fission products is a sophisticated process and calls for large and complex infrastructures, professional technical skills, and well-equipped laboratories. Only few centers worldwide are well-prepared to conduct this task (Ali and Ache, 1987; NAP, 2016, 2018; NEA, 2019). Furthermore, the generation of massive quantities of toxic radioactive waste containing medium and long-lived radionuclides has to be considered. A significant number of Curies (∼50 Ci) of fission radioactive waste are generated per production of one Curie (Ci) of ^99^Mo (Boyd, 1987; IAEA, 1998; Tur, 2000). The difficulties mentioned above are clearly reflected in the continuing need for long-term capital investments and routine operating expenditures. As a result, the production cost of 1 Ci of ^99^Mo from the fission route is four times higher than that of 1 Ci of neutron-capture-produced ^99^Mo (Boyd, 1982a).

#### 2.1.2 Neutron-capture-produced ^99^Mo

^99^Mo can also be produced through the neutron activation of natural or enriched ^98^Mo targets in the thermal flux of typical research reactors. This technology has been used for ^99^Mo production for more than five decades. Molybdenum has seven different naturally occurring stable isotopes, as shown in Table 6. ^98^Mo is the most naturally abundant molybdenum isotope with 24.13%.

**TABLE 6:** The naturally occurring molybdenum isotopes (Magill et al., 2022).

Isotope	Abundance, %	Thermal (n,γ) cross-section, barn	Half-life	Mode of decay	Decay product	Remark
^92^Mo	14.649	2E-7^a^	Stable	N.A	N.A	^92^Mo(n,γ)^93m^Mo
		0.08				^92^Mo(n,γ)^93g^Mo
^94^Mo	9.187	0.34	Stable	N.A	N.A	
^95^Mo	15.873	13.4	Stable	N.A	N.A	
^96^Mo	16.673	0.55	Stable	N.A	N.A	
^97^Mo	9.582	2.2	Stable	N.A	N.A	
^98^Mo	24.292	0.130	Stable	N.A	N.A	
^100^Mo	9.744	0.199	7.01E18 years	Double β^-^	^100^Ru (Stable)	The only unstable naturally occurring isotope of Mo

a This value is deduced from (Gedeonov and Nosov, 1989).

During the irradiation process, the target nucleus, ^98^Mo, captures one thermal neutron and emits gamma rays to form ^99^Mo according to the nuclear reaction M98o(n,γ)M98o . The produced ^99^Mo is a Carrier-Added (CA) product (not carrier-free) with low specific activity, as only a small portion of molybdenum is converted to ^99^Mo. In other words, the ^99^Mo activity is diluted with the inactive bulk of the ^98^Mo target (Molinski, 1982). The specific activity of the produced ^99^Mo can be determined from the following equation:

$$S=\frac{L\times \emptyset \times \sigma \times a}{3.7\times 10^{7}\times A}\left(1-e^{-\lambda t}\right)\;mCi/g$$ (1)

$$S=\frac{L\times \emptyset \times \sigma \times a}{A}\left(1-e^{-\lambda t\;}\right)\;Bq/g$$ (2)

Where;

**S** is the specific activity of the produced ^99^Mo in mCi/g or Bq/g,
**L** is the Avogadro number (6.022 × 10^23^),
**σ** is the thermal activation cross-section (0.13 barns, i.e., 0.13 × 10^–24^ cm^2^),

ϕ is the thermal neutron flux in n/cm^2^s,
**a** is the abundance of ^98^Mo isotope (∼24.14%),
**A** is the atomic mass of molybdenum (95.94),
**t** is the total irradiation time,
**λ** is the radioactive decay constant (ln2/T_1/2_),
**T**_**1/2**_ is the half-life of the ^99^Mo product (T_1/2_ = 65.94 h), and it is in the same unit as **t.**

Based on Eq 1, 2, the ^99^Mo specific activity at the End of Bombardment (EOB) mainly depends on some essential parameters, such as the value of the neutron flux in the irradiation channel, the effective cross-section, the irradiation period, and the ^98^Mo content in the irradiated target (Binney and Scherpdz, 1978; Hetherington and Boyd, 1999).

The use of enriched ^98^Mo target material provides an adequate opportunity to enhance the specific activity. This increase is proportional to the enrichment factor. Therefore, the specific activity yield of the produced ^99^Mo can be improved by a factor of four using enriched targets, which have a ^98^Mo content ≥96%.

^98^Mo targets can also be defined according to the chemical form of the target material, which involves the use of MoO_3_ or Mo metal. The irradiated MoO_3_ powder can be easily dissolved compared to Mo metal. However, when molybdenum metal is used, the amount of ^98^Mo in the same irradiation space is higher, resulting in higher ^99^Mo yields per Gram material.

One of the main parameters that limit the specific activity that results from this method is the small neutron capture cross-section of ^98^Mo targets, which is 0.13 barns for thermal neutrons. However, this value can be increased for the same target to 6.7 barns when the irradiation is conducted in the epithermal energy region (Lavi, 1978; IAEA, 2013a). In practice, specific activities of 10–15 Ci/g Mo have been achieved (Ryabchikov et al., 2004). The produced specific activity of ^99^Mo from neutron irradiation of ^98^Mo targets is particularly low compared to ^99^Mo provided by the uranium fission method (see Table 3). However, the specific activity can be increased by a factor of eight if an enriched ^98^Mo target of a particular geometry is irradiated in a high flux reactor (Ali and Ache, 1987; Hetherington and Boyd, 1999; Hasan and Prelas, 2020).

In principle, commercial power reactors offer an adequate neutron spectrum and neutron flux for producing Low Specific Activity (LSA) ^99^Mo. However, power reactors are usually not equipped for the introduction of samples for irradiation during operation. This fact significantly limits the use of power reactors as irradiation sources. There are exceptions. The new EPR reactors and the older KONVOI type Pressurized Water Reactors (PWR) have a built-in, so-called aeroball system for *in-situ* flux measurements. During operation, ^51^V spheres of 1.7 mm diameter can be filled in columns in the reactor, irradiated for a short time period, and extracted again. The produced activity is then measured on a so-called measuring table (Konheiser et al., 2016; Framatome, 2019a). This system could be repurposed to introduce metallic Mo-spheres or MoO_3_ ceramic spheres for irradiation (Framatome, 2019b). As the columns are several meters long and there are a large number of columns, sufficient amounts of Mo can be irradiated.

Furthermore, one of the potential projects that utilize neutron-capture-produced ^99^Mo has been implemented by Northstar Company in the United States. Northstar uses the Missouri University Research Reactor (MURR) to irradiate both natural and enriched ^98^Mo targets. The estimated production capacity of this project will be about 4500 six-day Curie ^99^Mo per week by 2024 (NEA, 2019). Northstar is supplying LSA ^99^Mo solution to be used with a generator system of RadioGenix, which produces ^99m^Tc in a computer-controlled generator system.

Clearly, the neutron activation technology eliminates proliferation concerns, as it offers a clean method to provide ^99^Mo without the need to handle any fissile materials. In addition, it asks for fewer technological requirements and financial budgets compared to the uranium fission approach (Boyd, 1982a). Moreover, based on the fact that only molybdenum targets are irradiated, this process involves less demanding chemical separation and purification steps as well as the generated radioactive waste level can be neglected. Furthermore, the good global distribution of reactors with high neutron flux capability could significantly support the ^99^Mo economy. The method is fast, and a time span of 3 days from the EOB to the arrival of the generator to the end-user is feasible. On the one hand, the ^99^Mo decay loss can be minimized, as the ^99^Mo activity can be delivered in a short period directly after production. On the other hand, a stable supply of ^99^Mo can be guaranteed even in case of any unplanned interruptions that may arise from the fission production line.

Despite the potential advantages of producing neutron-capture-produced ^99^Mo, its utility for the production of ^99m^Tc generators faces some potential challenges, especially with conventional alumina columns. Using such materials leads to a large elution volume with a very low ^99m^Tc radioactive concentration and a significant ^99^Mo breakthrough risk. Recently, extensive research efforts have been conducted for alternative strategies to overcome these problems. In this regard, the challenges and the current progress are therefore discussed in more detail in Sections 3.2, 3.3.

### 2.2 Accelerator-based ^99^Mo production

Recently, there has been a growing interest in using modern particle accelerators as a promising solution to produce ^99^Mo for medical applications. This approach allows the production of ^99^Mo without the need for HEU targets and generates relatively low amounts of radioactive waste compared to nuclear reactors (Van der Marck et al., 2010). The idea is built on the acceleration of charged particles, such as protons, deuterons, or electrons, to induce nuclear reactions in the target material. In some cases, the accelerated particles can interact with the target material and directly produce ^99^Mo or even ^99m^Tc. In other cases, the reaction becomes more complex and takes place in two steps. In the first step, the primary charged particles are successfully accelerated and strike an intermediate target material to produce secondary particles, such as neutrons or high-energy photons. Then, the secondary particles interact with the main target material to produce ^99^Mo (NEA, 2010). The choice of one of these techniques mainly depends on the post-irradiation ^99^Mo production capability. The primary accelerator-based ^99^Mo − or direct ^99m^Tc production approaches are summarized in Table 7. In Table 7, the irradiation yields were calculated under modern-day, realistic assumptions concerning beam intensities using publicly available nuclear codes. For photonuclear reactions, it was assumed that the electron beam is delivered by an IBA TT-300 HE rhodotron (IBA, 2018) impinging on a high-power converter target (Türler et al., 2021). The photon spectrum and the yield of ^99^Mo were calculated by Vagheian (Vagheian, 2022). For proton and deuteron-induced reactions, it was assumed that the beam is being delivered by a IBA Cyclone IKON-1000 cyclotron as a typical representative of accelerators in the 30 MeV energy range (IBA, 2022). It was assumed that a solid target station could accept a maximum proton beam current of 400 μA (i.e., Nirta High Power Solid target (90–400 μA)), since molybdenum is a very refractive element. For the calculations, the web-based “Medical Isotope Browser” was employed (see the footnote in Table 7). As can be seen from Table 7, the production of ^99^Mo with an electron accelerator from ^100^Mo appears to be most promising due to the large activities that can be produced. Proton irradiations of ^100^Mo yield significantly less yield of ^99^Mo, while producing more undesired by-products. The direct production of ^99m^Tc from ^100^Mo is a viable option but significantly reduces the distribution radius of the product (see Section 3.1). In the following, the accelerator-based production of ^99^Mo is discussed in more detail.

**TABLE 7:** The main accelerator-based ^99^Mo and ^99m^Tc production routes and their production capabilities per day.

Facility	Accelerated particle	Incident particle	Target material	Nuclear reaction	Reaction parameters			Thick target yield at EOB, Ci	Available activity at calibration, Ci
					Target thickness, mm	Incident energy, MeV	Incident current^a^, µA		
Electron accelerator	Electron	Photon	^nat.^U	^238^U(γ, f)^99^Mo	3.17^b^	40	3125	2.30^c^	1.08
	Electron	Photon	^100^Mo	^100^Mo(γ,n)^99^Mo	9.96^b^	40	3125	137^c^	64.34
Deuteron accelerator	Deuteron	Neutron	^100^Mo	^100^Mo(n,2n)^99^Mo	N.A	N.A	N.A	N.A	N.A
	Deuteron	Deuteron	^100^Mo	^100^Mo(d,p2n) ^99^Mo	0.17	15	100	0.02	0.01
					0.20	16	100	0.03	0.01
					0.24	17	100	0.05	0.02
					0.28	18	100	0.07	0.034
	Deuteron	Deuteron	^100^Mo	^100^Mo(d,3n)^99m^Tc	0.14	15	100	0.35	N.A
					0.18	16	100	0.67	N.A
					0.21	17	100	1.12	N.A
					0.25	18	100	1.73	N.A
Proton accelerator	Proton	Proton	^100^Mo	^100^Mo(p,pn)^99^Mo ^100^Mo (p,2p)^99^Nb →^99^Mo	0.30	15	400	0.26	0.12
					0.61	20	400	2.52	1.18
					0.99	25	400	7.36	3.46
					1.43	30	400	13.85	6.50
					1.92	35	400	21.44	10.07
	Proton	Proton	^232^Th	^232^Th(p,f)^99^Mo	N.A	N.A	N.A	N.A	N.A
	Proton	Proton	^100^Mo	^100^Mo(p,2n)^99m^Tc	0.32	15	400	21.67	N.A
					0.63	20	400	48.01	N.A
					1.01	25	400	62.23	N.A
					1.45	30	400	68.18	N.A
					1.94	35	400	73.04	N.A
	Proton	Proton	^98^Mo	^98^Mo(p,γ) ^99m^Tc	2.07	35	400	0.01	N.A
α-particle accelerator	α-particle	α-particle	^96^Zr	^96^Zr(α,n)^99^Mo	0.06	15	100	0.03	0.01
					0.11	20	100	0.04	0.02
					0.16	25	100	0.04	0.02
					0.22	30	100	0.04	0.02
					0.29	35	100	0.05	0.02

The data were calculated using “Medical Isotope Browser” of IAEA: https://www-nds.iaea.org/relnsd/isotopia/isotopia.html (accessed on 08.06.2022) utilizing recommended cross section data from (Tarkanyi et al., 2019).

a https://www.iba-radiopharmasolutions.com/cyclotrons-equipment (accessed on 08.06.2022).

b Thickness of 1 radiation length (1 X_0_).

c Yield of a cylindrical target of 1 X_0_ thickness and 2 cm diameter at a distance of 2 cm from a distributed Ta converter of 4.5 mm thickness. The thick target yield can be obtained by multiplication by a factor of about 4.

#### 2.2.1 Photonuclear reactions

With electron accelerators, a high-intensity electron beam is allowed to impinge on a dense target material to produce Bremsstrahlung photons. These photons can be used to produce ^99^Mo by two main strategies:

1- Photon-induced fission (photofission) reaction.
2- Photon-induced nuclear (photonuclear) reaction.

In the photo-fission method, high specific activity ^99^Mo can be obtained from the reaction U238(γ,f)M99o . The ^99^Mo production output is approximately 3700 times lower than the neutron-induced uranium fission method, attributed to the considerably lower reaction cross-section (0.16 barn) (van der Marck, 2010; Qaim, 2012). After the irradiation process is accomplished, ^99^Mo is chemically separated from the ^238^U targets in the same way as the previously described technique for irradiated HEU targets in nuclear reactors (Owen et al., 2014). For commercial production, high energy accelerators need to be used (Ruth, 2009). However, this option is still under demonstration. Using ^235^U targets increase the ^99^Mo yield (Krishichayan et al., 2017). Nonetheless, this option does not have enough justification due to higher production costs, high process complexity, and proliferation issues (Bertsche, 2010).

The photonuclear transmutation reaction offers a promising strategy for ^99^Mo production according to the reaction M100o(γ,n)M99o . The maximum cross-section of this reaction is 0.15 barn at a photon energy of 14 MeV (van der Marck, 2010; Crasta et al., 2011), which is larger than the ^238^U photo-fission reaction for producing ^99^Mo, which results in a higher ^99^Mo yield by a factor of about 17 in case highly enriched ^100^Mo targets are used (Bertsche, 2010). Specific activities of about 5 Ci/G of ^100^Mo can be achieved, resulting in production capacities of more than 100 Ci per day. Mang’era et al. (2015) reported the production of 2.4–3 GBq/h/g/mA ^99^Mo from 97.39% enriched ^100^Mo targets. Commercially available electron accelerators can deliver up to 3 mA of beam at energies up to 40 MeV. Also, a high-power converter target design has recently been submitted as patent application (Türler et al., 2021). Therefore, future production of 100 Ci per day with a specific activity of 5 Ci/g appears feasible. Nevertheless, the specific activity yield is still lower than for the U238(γ,f)M99o reaction. In order to achieve a sustainable long-term ^99^Mo supply, two main difficulties need to be urgently solved. First, while high power electron accelerators are commercially available, suitable converter targets that can accept more than 100 kW beam power still need to be developed (Türler et al., 2021). Second, the possibility of reusing the valuable ^100^Mo target material should receive intensive research consideration (Bertsche, 2010).

#### 2.2.2 14.1 MeV neutron-induced reactions

^99^Mo can also be produced through the bombardment of a ^100^Mo target with high energy neutrons according to the nuclear reaction ^100^ *Mo*(*n*,2*n*)^99^ *Mo*. Here, the 14.1 MeV neutrons are generated in the reaction ^3^ *H*(*d*,*n*)^4^ *He*. The reaction cross-section for the reaction ^100^ *Mo*(*n*,2*n*)^99^ *Mo* is about 1.5 barn at the neutron energy level of 14.1 MeV, which is ten times higher than the cross-section of the ^98^Mo(n,γ)^99^Mo reaction in the thermal region. In addition, a long irradiation period of more than 8 days and a high-intensity neutron flux ≥ 10^13^ n/cm^2^s is required (Nagai and Hatsukawa, 2009). Even so, the production of low specific ^99^Mo with large amounts of inactive carrier is still present even with ^100^Mo targets of 100% enrichment. Under the conditions mentioned above, the specific activity of the produced ^99^Mo was estimated at about 2 Ci/g (79 GBq/g).

In another approach, 14 MeV neutrons from a D-T generator are directed at a low enriched ^235^U salt solution, where ^99^Mo is produced as a fission product and periodically separated. This approach is being commercially exploited by Shine Medical Technologies Inc. (Wisconsin, United States) (Ruth, 2020).

#### 2.2.3 Spallation neutron-induced reactions

Similar to the concept of neutron-induced fission or capture reactions in a nuclear reactor, ^99^Mo can be produced with the use of an Accelerator-Driven Subcritical Reactor (ADSR) through ^235^ *U*(*n*,*f*)^99^ *Mo* and ^98^ *Mo*(*n*,*γ*)^99^ *Mo* reactions (Abderrahim et al., 2010; Bertsche, 2010; NEA, 2010; Owen et al., 2014; Syarip et al., 2018). However, the used neutrons have a different origin. High-intensity neutron flux can be generated following the bombardment of a heavy mass target material with an accelerated proton beam, such as lead, uranium, tungsten, or tantalum. This reaction produces fast neutrons with a kinetic energy level of more than 1 MeV up to almost the beam energy of the proton beam, which then is slowed down to the epithermal energy level with the help of a moderator. These conditions offer a unique opportunity to obtain a reasonable ^99^Mo yield with a relatively high specific activity using the ^98^ *Mo*(*n*,*γ*)^99^ *Mo* option. Uranium targets have to undergo routine chemical processing to provide purified M99oO42− active solution (Pillai et al., 2013).

#### 2.2.4 Other production methods

Many research groups have proposed other possibilities through proton- or deuteron-induced ^99^Mo production (Beaver and Hupf, 1971; Lagunas-Solar et al., 1991; IAEA, 1999; Hermanne et al., 2007). For example, the M100o(p,pn)M99o reaction has a small cross-section and yields low amounts of ^99^Mo. Meanwhile, the reaction M100o(d,p2n)M99o has a twofold higher reaction cross-section than the former. Nonetheless, both require high-power accelerators. Despite using enriched ^100^Mo targets and high-energy power accelerators, these two strategies seem to be not satisfactory for stable long-term production of ^99^Mo for medical use.

## 3 ^99m^Tc production and supply strategies: Challenges and progress

### 3.1 Cyclotron-produced ^99m^Tc

The severe ^99^Mo production shortage during the past few years, along with the widespread concerns of unexpected future ^99^Mo supply inadequacy, have generated a growing need for establishing an independent sustainable supply of ^99m^Tc. Consequently, many research projects have been established to promote the direct production of ^99m^Tc exploiting cyclotron production. The cyclotron-based ^99m^Tc production can provide an independent option for domestic utilization (IAEA, 1999; Ruth, 2009, 2014; Gagnon et al., 2011; Hanemaayer et al., 2014; Owen et al., 2014; Metello, 2015; Boschi et al., 2017; Martini et al., 2018).

Several approaches have been proposed for direct ^99m^Tc production, such as ^100^ *Mo*(*p,2n*)^99m^ *Tc*, ^98^ *Mo*(*p,γ*)^99m^ *Tc*, ^100^ *Mo*(*d,3n*)^99m^ *Tc*, ^98^ *Mo*(*d,n*)^99m^ *Tc*, ^97^ *Mo*(*d,γ*)^99m^ *Tc*, and ^96^ *Mo*(*α,p*)^99m^ *Tc*. It is pertinent to point out that the proton-induced reactions produce the highest production yields compared to deuteron or alpha particle-induced reactions. In addition, accelerators with high beam intensity of deuterons or alpha particles are currently rare. For these reasons, they received less attention (Lagunas-Solar et al., 1991; IAEA, 1999, 2017; Takács et al., 2003; Guerin et al., 2010; Owen et al., 2014; Stolarz et al., 2015).

The direct production of the ^99m^Tc using energetic proton beams involves two reactions; M98o(p,γ)T99mc and M100o(p,2n)T99mc . The former has a very small reaction cross-section and produces a low ^99m^Tc yield, which is not convenient for large-scale production (Lagunas-Solar et al., 1991; Takács et al., 2003). The M100o(p,2n)T99mc is more promising for large-scale production because it has a higher cross-section, and its optimum proton energy range is compatible with most conventionally used cyclotrons. Therefore, it has been attracting considerable attention from the scientific community (Lagunas-Solar et al., 1991; Takács et al., 2003; Martini et al., 2018). This approach was first mentioned by Beaver and Hupf at the University of Miami School of Medicine in 1971 using enriched ^100^Mo targets (Beaver and Hupf, 1971). Afterward, the possibility of using natural ^100^Mo targets was evaluated by Almeida and Helus in 1977 (Lagunas-Solar et al., 1991; IAEA, 1999). Recently, many attempts have been conducted to explore the most appropriate conditions to produce considerable amounts of high specific activity of ^99m^Tc with the least possible impurity level. These studies concluded that the final ^99m^Tc product greatly depends on three primary factors: the ^100^Mo target enrichment quality, the irradiation conditions, and the duration of the chemical processing.

The most commonly used molybdenum target material is the Mo metal. In addition, the utilization of MoO_3_ and Mo_2_C is also possible (Richards et al., 2013). The fabrication of the molybdenum targets can be developed with the help of several technical advances. These approaches involve preparing foils, pressing and thermal sintering of molybdenum powder, vacuum sputtering technology, laser beam pressed molybdenum powder method, electrochemical plating technique, and electrophoretic deposition followed by thermal sintering process (Richards et al., 2013; Hanemaayer et al., 2014; Stolarz et al., 2015; Hou et al., 2016; Martini et al., 2018). The target purity plays a crucial role and greatly affects the quality of the final ^99m^Tc product. The contribution of other molybdenum isotopes with ^100^Mo in the target material leads to producing different impurities such as Tc, Nb, Ru, and Zr (Beaver and Hupf, 1971; Lagunas-Solar et al., 1991; IAEA, 1999; Guerin et al., 2010; Owen et al., 2014; Hou et al., 2016). To reduce the impurity level, the isotopic abundance of ^100^Mo targets must not be less than 99.5%. Some of the produced impurities can be removed during the separation and purification processes. However, the different Tc isotopes have the same chemical behavior as ^99m^Tc. Therefore, the separation of these isotopes poses considerable laborious challenges, especially with current conventional separation technology. The Tc isotopes can be classified into two groups. The first group involves the long-lived radioisotopes, such as ^99g^Tc (T_1/2_ = 2.11×10^5^ y), ^98^Tc (T_1/2_ = 4.2×10^6^ y), and ^97g^Tc (T_1/2_ = 4.21×10^6^ y). These radioisotopes decrease the final specific activity of the product, but they may not be involved in an additional dose for the patient (Beaver and Hupf, 1971; Lagunas-Solar et al., 1991). On the other hand, the second group contains the shorter half-lives radioisotopes, such as ^93m^Tc (T_1/2_ = 43.5 min), ^93g^Tc (T_1/2_ = 2.75 h), ^94m^Tc (T_1/2_ = 52 min), ^94g^Tc (T_1/2_ = 4.9 h), ^95m^Tc (T_1/2_ = 61 d), ^95g^Tc (T_1/2_ = 20 h), ^96m^Tc (T_1/2_ = 51.5 min), ^96g^Tc (T_1/2_ = 4.28 h), and ^100^Tc (T_1/2_ = 15.8 s). This group not only substantially drops the specific activity of the final product, but also delivers up to 30% extra unjustified dose to the patient (Beaver and Hupf, 1971; Lagunas-Solar et al., 1991; IAEA, 1999, 2017; Hou et al., 2012; Lededa et al., 2012; Owen et al., 2014; Meléndez-Alafort et al., 2019). Accordingly, the optimization of irradiation parameters offers another opportunity to minimize the produced technetium impurities. These parameters include the bombarding beam energy, irradiation time, and incident beam intensity.

As a generic rule, the higher the proton energy and the longer irradiation time, the higher the production yield. However, these two factors have to be well-controlled to reduce the co-production of undesirable impurities, resulting in a high level of specific activity product obtained. The threshold proton energy for the reactions M100o(p,2n)T99mc , M100o(p,3n)T98c , and M100o(p,4n)T97gc are 7.7, 17, and 24 MeV, respectively. In addition, further side reactions can take place in the energy range above 24 MeV. Therefore, the optimum bombarding energy is preferably maintained at an energy level lower than 24 MeV and applies the shortest possible irradiation time that can satisfy the production demand plan (Beaver and Hupf, 1971; Esposito et al., 2013; IAEA, 2017, 1999).

In order to achieve a high production yield, the beam power intensity should be set to the highest possible value. This beam current crucially depends on the target material, the applied incident beam angle, and the cooling system during the irradiation process (Beaver and Hupf, 1971; IAEA, 1999). It is worth mentioning that molybdenum targets have preferable chemical and physical properties reflected by their high melting point and excellent thermal conductivity. Therefore, they can withstand high beam currents. The target cooling process is also influenced by the thermal conductivity of the target material and the temperature of the used cooling item. Effective target cooling can be achieved by applying helium flow on the front side and water-cooling of the supporting material of the target during the irradiation process. Some research studies proposed the use of liquid nitrogen instead of water-cooling for more effective target cooling (IAEA, 2017, 1999).

After completing the irradiation process, the target is left to cool down to reduce the incorporation of short-lived impurities in the final product, followed by chemical processing. This step is crucial and should be achieved in the shortest possible time to minimize ^99m^Tc decay loss (Qaim, 2012). The post-production target processing can be achieved in two steps: target dissolution and chemical separation and purification. The efficient dissolution of the molybdenum targets is paramount for an effective ^99m^Tc recovery. The target dissolution can be performed either by chemical or electrochemical procedures. The chemical dissolution can be performed with the help of concentrated sodium hydroxide, hydrogen peroxide, and heating. In the case of foil targets, concentrated acids can also be used. The electrochemical dissolution system consists of the molybdenum target as an anode, a platinum layer as a cathode, and potassium hydroxide, which works as an electrolyte. Electrochemical methods may involve violent reactions and need double the time to achieve the same task compared to chemical methods (Gumiela, 2018; IAEA, 2017, 1999).

Many potential techniques have been implemented for the ^99m^Tc recovery from dissolved ^100^Mo targets. Because of the short half-live of ^99m^Tc, a fast separation reaction is a key to selecting the extraction method. The most commonly used methods include solvent extraction, which can be carried out with the use of Methyl Ethyl Ketone (MEK) or Cetyl Trimethyl Ammonium Bromide (CTAB) (Martini et al., 2018). Column chromatographic separation methods involve the use of ion exchanger sorbent materials with high radiation resistance, such as Dowex-1 or Polyethylene Glycol (PEG). Some other techniques have also been proposed, such as dry thermo-chromatographic extraction and the chemical precipitation of the molybdenum as a hetero-poly acid salt. The former generates small quantities of waste, and the latter could provide a reasonable separation yield. Nevertheless, both strategies are not widely utilized (IAEA, 1999; Boschi et al., 2017; Gumiela, 2018).

Based on these discussions and as mentioned earlier, the cyclotron-produced ^99m^Tc technology has been extensively studied in recent years, and some success has been achieved. Therefore, this method can be regarded as a backup solution for ^99m^Tc supply to deal with emergencies. However, its potential is inadequate to replace the current ^99^Mo/^99m^Tc generator production completely; as the following critical issues still call for convincing answers:

• Only for regional consumption: The short half-life of ^99m^Tc (T_1/2_ = 6.01 h) restricts its wide-scale distribution. As a result, the delivery advantage is only limited to hospitals located near the production sites. Therefore, the implementation of this supply strategy necessarily asks for an even geographic distribution of cyclotrons.
• High cost per dose: The end-user should receive the required dose at a reasonable price, and the dose price should be competitive with that delivered from a ^99^Mo/^99m^Tc generator. The use of enriched ^100^Mo targets to ensure the production of high^-^quality ^99m^Tc increases the production cost. In addition, the recycling process of the used target material adds an extra production expenditure. Furthermore, the long post-irradiation processing step and the transportation requirements are reflected by the loss of ^99m^Tc radioactivity. All these parameters can be translated into a significant cost factor.
• A low specific activity product delivers extra radiation dose to the patients: The utilization of highly enriched ^100^Mo targets is vital to produce ^99m^Tc with high specific activity suitable for imaging applications. A 100% isotopic purity of ^100^Mo targets is hard to achieve, and small quantities of other stable Mo isotopes are constantly incorporated into the target material. Consequently, considerable amounts of different technetium isotopes are always co-produced. On the one hand, these impurities reduce the specific activity of the produced ^99m^Tc product, which significantly affects the labeling efficacy of ^99m^Tc-radiopharmaceutical kits. On the other hand, it may contribute to additional radiation exposure to the patient (Hou et al., 2012; Lededa et al., 2012; Selivanova et al., 2015; Meléndez-Alafort et al., 2019).
• Uncertainty of long-term supply capability: The direct production of ^99m^Tc has been proposed since 1971, and it has not been thoughtfully implemented as a long-term supply strategy. Many recent efforts have focused on establishing a large-scale ^99m^Tc supply on a daily routine basis. However, several problems need to be solved. These challenges can be briefly summarized in the need for a sufficient daily availability of patient doses at a reasonable price. Additionally, this strategy continues asking for a realistic supply plan design that can deal with the production difficulties and find innovative logistic solutions to meet the rapid product delivery requirements.

### 3.2 ^99^Mo/^99m^Tc generators

Despite the fast global spread of diagnostic scans using ^99m^Tc-radiopharmaceuticals, the ^99m^Tc availability at the hospitals and nuclear pharmacies continues to be a potential challenge. These challenges include the production difficulties, notable price, and remarkable radioactivity loss due to frequent deliveries to the usage sites. Accordingly, its effective utilization is only restricted to sites with a well and close connection to the production centers (Sakr et al., 2017). In the light of this perceived need, different ^99^Mo/^99m^Tc generator strategies have been proposed and developed. ^99m^Tc generators are considered the most widely used strategy to ensure the ease of ^99m^Tc availability in a cost-efficient manner. In other words, it allows conducting a variety of ^99m^Tc scans independently of on-site production requirements (Knapp and Mirzadeh, 1994; Chakravarty et al., 2012a, 2012b; Knapp and Baum, 2012; Chakravarty and Dash, 2013; Sakr et al., 2017).

#### 3.2.1 ^99^Mo/^99m^Tc radioactive equilibrium in the generator system

The ^99^Mo/^99m^Tc generator technology has been developed to offer an easy and repetitive on-site supply of ^99m^Tc at desirable time intervals. Historically, ^99^Mo/^99m^Tc generators were named “cows”, from which a fresh ^99m^Tc radioactivity can be periodically “milked” or extracted. Based on the fact that ^99^Mo and ^99m^Tc are radioisotopes of two different elements, they have unique differences in their chemical and physical properties, and the generator system can supply ^99m^Tc with high specific activity, i.e., Non-Carrier-Added grade (NCA) and with high radionuclidic purity acceptable for radiopharmaceutical preparations (Lebowitz and Richards, 1974; Molinski, 1982; IAEA, 2013b).

^99^Mo/^99m^Tc generators are efficient radiochemical separation tools, by which a convincingly purified yield of the daughter, ^99m^Tc, can be effectively extracted from the decay of the parent ^99^Mo. In other words, the ^99^Mo/^99m^Tc generator concept is based on housing ^99^Mo to decay. Then, a complete separation of the generated ^99m^Tc can be performed without any disturbance of ^99^Mo. This separation process is managed through the ^99^Mo-decay/^99m^Tc-growth concept (Lebowitz and Richards, 1974; Molinski, 1982; Guillaume and Brihaye, 1986; Saha, 1992; Osso and Knapp, 2011; Knapp and Baum, 2012; Chakravarty and Dash, 2013; Gumiela, 2018).

**FIGURE 4:**
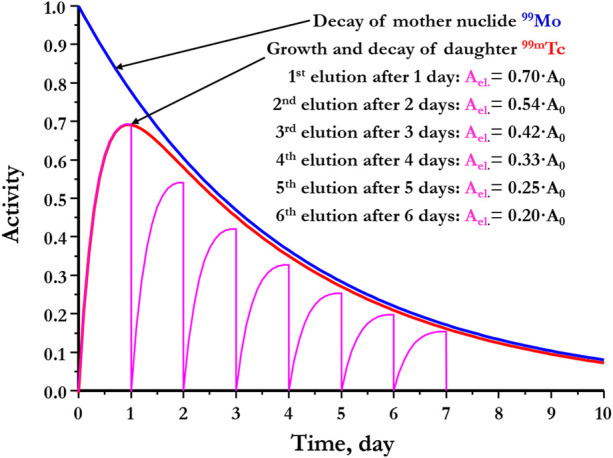
Transient radioactive equilibrium in ^99^Mo/^99m^Tc generator system. A_el_: Eluted ^99m^Tc activity. A_0_: ^99^Mo activity at calibration.

Generally, the generator concept is based on the parent/daughter relationship, by which the parent, with a longer half-life, decays to yield its shorter-lived daughter. Hence, a sort of radioactive equilibrium is established. This state is called secular or transient equilibrium, depending on the ratio between the two physical half-lives of the parent and its daughter. The secular equilibrium arises when the parent’s half-life is 100 times longer than the half-life of the daughter. Whereas the transient equilibrium, as in the ^99^Mo/^99m^Tc generator system, is recognized when the parent’s half-life is ten-fold longer than that of the daughter (Sakr et al., 2017). Figure 4 illustrates the transient equilibrium between ^99^Mo and ^99m^Tc.

The equilibrium between ^99^Mo and ^99m^Tc is reached after approximately four elapsed half-lives of ^99m^Tc. During the ^99m^Tc ingrowth, its production is remarkably faster than its decay. After the equilibrium is established, the ^99m^Tc growth and decay rates are equal. At this point, ^99m^Tc apparently decays with the longer half-live of ^99^Mo. This advantage considerably reduces the ^99m^Tc radioactivity loss during shipment to remote users. However, it decays with its physical half-life directly after its elution from the generator. Directly after the elution, the generator starts the same process from the beginning, as ^99^Mo decays to give rise to new fresh ^99m^Tc. Accordingly, ^99m^Tc begins to be re-built-up again (Lebowitz and Richards, 1974; IAEA, 2013b). The growth, extraction, and regrowth of ^99m^Tc are non-stop procedures. Consequently, the elution step can be repeated as long as sufficient ^99^Mo radioactivity is available to conduct the required applications. Therefore, ^99m^Tc can be eluted from the generator at periodical elution batches.

The mathematical correlation that describes the ^99m^Tc growth from the decay of ^99^Mo can be explained using the Batemann equations, as follows (Hidalgo et al., 1967; Lebowitz and Richards, 1974; Lamson et al., 1976; Ekelman and Coursey, 1982; IAEA, 2013b):

The decay of ^99^Mo can be expressed as

$$\frac{dN_{1}}{d_{t}}\;=-\;\lambda_{1}N_{1}$$ (3)

$$N_{1}\;=\;N_{1}^{{}^{\circ}}e^{-\lambda_{1}t}$$ (4)

Where:

λ: is the ^99^Mo decay constant,

$N_{1}$ : indicates the number of ^99^Mo atoms at the time (t).

$N_{1}^{{}^{\circ}}$ : refers to the number of ^99^Mo atoms when t = 0.

The production of ^99m^Tc is the same as the decay of ^99^Mo. Considering that ^99m^Tc decays at a rate of. Hence, the ^99m^Tc growth rate can be represented by:

$$\frac{dN_{2}}{d_{t}}=\;\lambda_{1}N_{1}-\lambda_{2}N_{2}=\;\lambda_{1}N_{1}^{{}^{\circ}}e^{-\lambda_{1}t}-\;\lambda_{2}N_{2}$$ (5)

$$∴N_{2}=\;\frac{\lambda_{1}}{\lambda_{2}-\lambda_{1}}N_{1}^{{}^{\circ}}\left(e^{-\lambda_{1}t}-e^{-\lambda_{2}t}\right)+N_{2}^{{}^{\circ}}e^{-\lambda_{2}t}$$ (6)

∵ If only ^99^Mo is present initially

$$∴\;N_{2}^{{}^{\circ}}=0$$

Accordingly, the Eq 6 can be reformulated as:

$$∴N_{2}=\;\frac{\lambda_{1}}{\lambda_{2}-\lambda_{1}}N_{1}^{{}^{\circ}}\left(e^{-\lambda_{1}t}-e^{-\lambda_{2}t}\right)$$ (7)

Taking into account that only about 87.5% of ^99^Mo decays to ^99m^Tc (IAEA, 2017).

$$∴N_{2}=\;0.875\;N_{1}^{{}^{\circ}}\frac{\;\lambda_{1}}{\lambda_{2}-\lambda_{1}}\left(e^{-\lambda_{1}t}-e^{-\lambda_{2}t}\right)$$ (8)

In order to give units of disintegration per second (A) rather than atoms (N), both sides of Eq 8 are multiplied by $\lambda_{2}$

$$∴N_{2}\lambda_{2}=\;0.875\;N_{1}^{{}^{\circ}}\frac{\;\lambda_{1}\lambda_{2}}{\lambda_{2}-\lambda_{1}}\left(e^{-\lambda_{1}t}-e^{-\lambda_{2}t}\right)$$ (9)

$$∵A_{2}=N_{2}\lambda_{2},\;A_{1}^{{}^{\circ}}=N_{1}^{{}^{\circ}}\lambda_{1}$$

$$∴A_{2}=\;0.875\;A_{1}^{{}^{\circ}}\frac{\;\lambda_{2}}{\lambda_{2}-\lambda_{1}}\left(e^{-\lambda_{1}t}-e^{-\lambda_{2}t}\right)$$ (10)

At the time (t), when the radioactive equilibrium between ^99^Mo and ^99m^Tc activities is reached, the growth rate of ^99m^Tc is equal to its decay rate:

$$∴\;\frac{dA_{2}}{d_{t}}=0$$

$$∴\;\frac{dA_{2}}{d_{t}}=\left(\frac{0.875\;A_{1}^{{}^{\circ}}\lambda_{2}\lambda_{2}}{\lambda_{2}-\lambda_{1}}\right)e^{-\lambda_{2}t}-\left(\frac{0.875\;A_{1}^{{}^{\circ}}\lambda_{1}\lambda_{2}}{\lambda_{2}-\lambda_{1}}\right)e^{-\lambda_{1}t}\;=0$$ (11)

$$∴\left(\frac{0.875\;A_{1}^{{}^{\circ}}\lambda_{2}\lambda_{2}}{\lambda_{2}-\lambda_{1}}\right)e^{-\lambda_{2}t}=\left(\frac{0.875\;A_{1}^{{}^{\circ}}\lambda_{1}\lambda_{2}}{\lambda_{2}-\lambda_{1}}\right)e^{-\lambda_{1}t}$$ (12)

$$∴\;\frac{e^{-\lambda_{1}t}}{e^{-\lambda_{2}t}}=\frac{\lambda_{2}}{\lambda_{1}}$$ (13)

$$∴\;e^{t\left(\lambda_{2}-\lambda_{1}\right)}=\frac{\lambda_{2}}{\lambda_{1}}$$ (14)

$$∴t\left(\lambda_{2}-\lambda_{1}\right)=\ln\frac{\lambda_{2}}{\lambda_{1}}$$ (15)

Hence, the maximum activity of the ^99m^Tc, which is present at the time (t), can be calculated from the following equation:

$$T_{\max}=\frac{1}{\lambda_{2}-\lambda_{1}}\ln\frac{\lambda_{2}}{\lambda_{1}}$$ (16)

$$∵\lambda_{1}=\frac{\ln2}{{\text{T}}_{1/2,\;1}}\;=\;\frac{0.693}{65.94\;h}\;=0.01051\;h^{-1}$$ (17)

$$∵\;\lambda_{2}=\frac{\ln2}{{\text{T}}_{1/2,\;2}}\;=\;\frac{0.693}{6.01}=0.1153\;h^{-1}$$ (18)

$$∴\;T_{\max}=22.9\;h$$

Clearly, the radioactive relationship between ^99^Mo and ^99m^Tc offers an excellent opportunity to introduce an ideal generator system, by which the separation of the short-lived ^99m^Tc at favorable time intervals can easily be achieved. The eluted radioactivity can be quantified using equation number (6). Nevertheless, it is significantly influenced by the elution process efficiency. Hence, the maximum ^99m^Tc radioactivity can be eluted on a daily basis, which is one of the potential advantages of the generator system.

#### 3.2.2 Criteria of a clinical ^99^Mo/^99m^Tc generator

The effective ^99^Mo/^99m^Tc generator system should fulfill the following characteristics (Lebowitz and Richards, 1974; Sakr et al., 2017; IAEA, 2018):

• Use simple, safe, and user-friendly operational protocols that avoid any violent chemical reactions and support a feasible implementation at hospitals and nuclear medicine centers.
• Assure rapid radiochemical separation to minimize the ^99m^Tc radioactivity decay loss.
• Provide the highest possible elution yield with a reproducible separation efficiency over the generator operation lifetime.
• Capable of sustainable elutions with a negligible amount of radioactive waste.
• Provide a carrier-free ^99m^Tc radioactivity with high specific activity without any need for purification processes.
• Supply sterile, isotonic, and pyrogen-free ^99m^Tc radioactive solution at any elution cycle without any further need for chemical treatment.
• Produce ^99m^Tc with high radioactive concentration adequate for the radiopharmaceuticals synthesis without any need for post-elution concentration steps.
• Supply the ^99m^Tc radioactivity with high chemical, radiochemical, and radionuclidic purities. The chemical purity expresses how much of the produced ^99m^Tc is free from inactive impurities that arise from the target material and/or the used chemicals. The radiochemical purity is the percentage of ^99m^Tc radioactivity present in the pertechnetate chemical form, ^99m^TcO_4_^-^. The radionuclidic purity represents the percentage of the ^99m^Tc radioactivity to the total radioactivity of its solution.
• Minimize the personnel involved during the elution process to reduce the radiation exposure dose.
• Involve the use of chemical solutions and materials with high radiation stabilities to avoid any possible contamination to ^99m^Tc radioactivity.

#### 3.2.3 Production technologies of ^99^Mo/^99m^Tc generators

Historically, the idea of a ^99^Mo/^99m^Tc generator system was first proposed in the middle of the 1950_S_ during the development of ^132^Te/^132^I generators at Brookhaven National Laboratory (BNL). In this experiment, the ^99m^Tc was detected as a trace impurity in the ^132^I eluted solution. Afterward, it was understood that this ^99m^Tc was produced from its parent, ^99^Mo, which took the same separation path of ^132^Te throughout the chemical processing of the fission products. This unique parent-daughter chemistry between ^99^Mo and ^99m^Tc has encouraged the fabrication of the first ^99^Mo/^99m^Tc generator (Richards et al., 1982; Anderson et al., 2019). In 1957, the first ^99^Mo/^99m^Tc generator was produced at BNL (Tucker et al., 1958; Molinski, 1982; Richards et al., 1982). Then, in early 1960, the first ^99^Mo/^99m^Tc generator was used for clinical research at Brookhaven medical department, which paved the way for the first utilization of ^99^Mo/^99m^Tc generators in clinical investigations at Argonne Cancer Research Hospital in 1961 (Richards, 1965; Lebowitz and Richards, 1974; Atkins, 1975). Later on, through the following seven decades, the idea was developed, and several ^99^Mo/^99m^Tc generator structures were established based on the different physical and chemical behaviors of ^99^Mo and ^99m^Tc radionuclides (Allen, 1965; Boyd, 1987; IAEA, 1995, 2013b; Maoliang, 1997; Osso and Knapp, 2011; Knapp and Baum, 2012; Mostafa et al., 2016).

The careful selection of the radiochemical separation strategy is considered the cornerstone for the fundamental development of ^99m^Tc generators. In fact, this selection focuses explicitly on simplifying the extraction process and avoiding any technical complexities for the users. In addition, it supplies the possible maximum ^99m^Tc yield with the minimum quantity of radioactive waste. Furthermore, it supports a rapid ^99m^Tc elution with high-performance quality, which consequently; can be easily adopted as a mature commercial technology. In the light of this context, the following subsidiary sections underline the critical features of potential ^99^Mo/^99m^Tc generators production strategies. Table 8 highlights a comparison between the potential development technologies of ^99m^Tc radioisotope generators.

**TABLE 8:** The potential production strategies of ^99^Mo/^99m^Tc generators.

Production technology				Separation technique	Advantages	Disadvantages
Column chromatography	Based on alumina	Using (n,f)^99^Mo		Selective sorption of ^99^Mo (solid-phase extraction)	• Well-established technology	• Increases the nuclear proliferation concerns
					• Produces clinical-grade ^99m^Tc in high radionuclidic, radiochemical, and chemical purities	• Produces large quantities of long-lived radioactive wastes
					• Supplies ^99m^Tc in high RAC	
	Based on gel matrices	Using (n,γ)^99^Mo			• Eliminates proliferation concerns	• Supply ^99m^Tc with very low RAC
						• Retention of ^99m^Tc on the column matrix
						• Production of undesirable radioactive impurities
						• A multi-step procedure leads to the loss of the ^99^Mo radioactivity
	Based on nano-sorbents		Using (n,γ)^99^Mo		• Eliminates proliferation concerns	• Not yet demonstrated at activity scale necessary for clinical use
					• The production of a ^99m^Tc with desirable RAC	• Is not currently standard practice in ^99^Mo/^99m^Tc generators
					• Improves ^99^Mo economy	
					• Inexpensive	
Sublimation				Differences in the sublimation temperatures of ^99^Mo and ^99m^Tc oxides	• Generates a ^99m^Tc radionuclide of high purity	• Complex procedure
						• Require high degree of safety standards
						• Low ^99m^Tc separation efficiency yield from ^99^Mo
Solvent extraction				Difference in the solubility of ^99^Mo and ^99m^Tc in two immiscible liquid phases	• Low stability of the organic solvents
					• Low separation efficiency
					• Requires a very high degree of radiation safety considerations
Electrochemical				Difference in the reduction potential of ^99^Mo and ^99m^Tc	• The capacity is not limited by the amount of sorbent or extractant	• Expensive
						• Generate H_2_ and O_2,_ leading to explosion danger
Supported Liquid Membrane (SLM)				Selective extraction of the ^99m^Tc using porous hydrophobic membrane		• Slow separation kinetics
						• Unsatisfactory ^99m^Tc yield
						• ^99^Mo breakthrough
						• Radiation instability of the membrane and the extractant
						• Generates significant amounts of radioactive waste

##### 3.2.3.1 Sublimation generators

Sublimation is a physical phenomenon, which can be defined as the direct conversion of solids into a gas without moving through the liquid state. Perrier and Segrè were the first who described the possibility of a ^99m^Tc thermal extraction from ^99^Mo based on the difference in the sublimation temperatures or volatilities of their oxides (Perrier and Segrè, 1937). Tc_2_O_7_ and MoO_3_ become volatile at temperatures of 550 and 1,000°C, respectively (Perrier and Segrè, 1937; Childs, 2017). The separation steps were reported through several research studies. The irradiated ^99^MoO_3_ target is carefully placed inside a muffle furnace, in which a flow of oxygen stream is allowed to pass through. Then, the temperature is gradually elevated to about 800 °C. On heating, Tc_2_O_7_ is formed, which sublimates at 550°C. After that, the volatile Tc_2_O_7_ vapors are carried by the oxygen stream and trapped using a cooled surface. Finally, they are allowed to be completely dissolved in water or saline solution to obtain ^99m^TcO_4_^−^ (Hallaba and El-Asrag, 1975; Molinski, 1982; Zsinka, 1987; Miller, 1995; Christian et al., 2000; IAEA, 2013b).

This generator strategy generates a small volume of radioactive waste. In addition, it provides high specific activity ^99m^Tc even if ^99^Mo of low specific activity origin is available. However, attempts to implement this technique in nuclear medicine departments as a clinical-based generator system suffered from several critical constraints. For instance, it includes the manipulation of bulk, complex, and fragile equipment, which needs a meticulous operational methodology and an advanced level of precautions and safety standards. Moreover, the ^99m^Tc separation yield is very low. Furthermore, it uses high heating temperatures, which may lead to a serious radiation contamination risk in case of any operational failure. Accordingly, these potential drawbacks discouraged further development plans in this direction.

##### 3.2.3.2 Solvent extraction generators

The solvent extraction-based generator is a multi-step separation technique by which ^99m^TcO_4_^-^ can be recovered from an aqueous solution that contains the ^99^Mo/^99m^Tc mixture by the extraction into an organic solvent (IAEA, 2013b). The separation of ^99m^Tc from its parent with the help of organic extractants was originally demonstrated by Gerlit in 1956 (Gerlit, 1956; Anders, 1960; Lebowitz and Richards, 1974; Molinski, 1982).

The separation process involves the dissolution of the irradiated ^99^Mo target in an alkaline solution. Then, an organic solvent is added, which is followed by multiple-extraction cycles. Each cycle includes a vigorous shaking of the two immiscible phases to allow the ^99m^TcO_4_^−^ to migrate from the original aqueous solution to the organic extractant phase leaving its parent behind. After that, the extractant is segregated, washed several times, and thermally evaporated. Finally, the residual precipitate is redissolved in a saline solution. The process efficiency strongly depends on the selective capability of the extractant solvent for the ^99^Mo/^99m^Tc pair. In other words, the process relies on the relative distribution or solubility ratio of the two radionuclides between the extractor and the original aqueous solution (Chattopadhyay et al., 2010, 2014; IAEA, 2013b; Mitra et al., 2020).

DM99o =(CM99o)extractant(CM99o)aqueous (19)

DT99mc =(CT99mc)extractant(CT99mc)aqueous (20)

Therefore, the ideal extractant should show a considerable selective affinity towards ^99m^Tc and neglected distribution selectivity for ^99^Mo. The first used organic extractant to achieve the extraction process was Methyl Ethyl Ketone (MEK). After that, various organic solvents were reported (Allen, 1965; Anwar et al., 1968; Baker, 1971; Tachimori et al., 1971; Iqbal and Ejaz, 1974; Noronha, 1986; Bhatia and Turel, 1989; Bhatia and Turel, 1989; Minh and Lengyel, 1989; Taskaev et al., 1995; Chen and Tomasberger, 2001; Zykov et al., 2001; Skuridin and Chibisov, 2010). Even so, MEK is still considered the predominant extractant of choice because of its highly selective behavior for ^99m^TcO_4_^-^, which permits the production of ^99m^Tc with high specific activity (IAEA 2017).

Practically, the solvent extraction generators are associated with a number of serious drawbacks that impede their wide-scale utilization; it is a complicated multi-step process with low extraction efficiency and requires a long separation time, resulting in the loss of ^99m^Tc radioactivity. Moreover, the separation step consumes large volumes of the organic extractant, creating significant radioactive waste. In addition, a strong possibility of fire hazards that result from the flammable nature of MEK. Furthermore, the poor radiation stability of organic solvents may heighten the contamination risk of the final ^99m^Tc product with some organic impurities.

##### 3.2.3.3 Electrochemical generators

The electrochemical-based ^99m^Tc generator can be introduced as a separation technique built on an oxidation-reduction reaction. This reaction is mainly governed by the difference in the formal reduction potential of the ^99^Mo/^99m^Tc pair. Herein, the generator system consists of an electrochemical cell, in which the ^99m^Tc radionuclide can be separated from the ^99^Mo/^99m^Tc equilibrium mixture by a reduction reaction on the surface of an electrode under the effect of an external voltage in accordance with its electrochemical reduction potential, i.e., electrochemical deposition. The ^99^Mo/^99m^Tc pair exhibits different electrochemical reduction potentials, and their electrochemical reduction reactions can be illustrated as follows (Chakravarty et al., 2012b; 2012c, 2011, and 2010a):

$$MoO_{4}^{2-}+4\;H_{2}O+6e^{-}\;\to Mo\;+8\;OH^{-}\;E^{o}=-1.05\;V$$ (21)

$$TcO_{4}^{-}+4\;H^{+}+3\;e^{-}\;\to TcO_{2}\;+2\;H_{2}O\;E^{o}=+0.738\;V$$ (22)

It can be observed that the ^99m^TcO_4_^−^ species have a higher electrochemical reduction potential than the ^99^MoO_4_^2-^ ions, which offers a positive advantage in facilitating the separation step.

The process starts with the dissolution of the irradiated ^99^Mo target in a strong alkaline electrolyte (pH ∼ 13). Then, a constant potential is applied and adjusted for a certain period to permit the ^99m^TcO_4_^-^ species to be electro-deposited on the surface of the cathode electrode. After that, this cathode is removed from the working cell and transferred to a new cell, where the deposited ^99m^Tc can be stripped back and redissolved in a saline solution by applying a high reverse potential (reverse electrode polarity) for a short period. By this technique, ^99m^Tc can be oxidized back to the solution in the form of ^99m^TcO_4_^-^. Eventually, ^99m^Tc eluate is allowed to pass through an alumina column to minimize the ^99^Mo contamination level.

In order to achieve a satisfactory ^99m^Tc separation yield, some parameters have to be well-optimized during the electrolysis process. For instance, careful attention should be devoted to adjusting the effective potential, proper selection of suitable electrodes and electrolysis medium candidates, and successful control of the electrolyte pH and separation time.

The applied potential is the crucial factor for a successful extraction. Generally, the electrochemical separation can be performed by either applying a constant current (galvanostatic) or a constant potential (potentiostatic) conditions. However, the potentiostatic condition offers favorable ^99m^Tc deposition and, in the meantime, limits the precipitation of ^99^Mo and/or any other contaminants. The electrolysis potential should be adjusted to be more negative than the formal reduction potential of ^99m^TcO_4_^-^ species and less negative than the electrochemical reduction potential of ^99^MoO_4_^2-^ ions. Therefore, the optimum reduction potential for the ^99m^Tc generator system should be in the region of:

−1.05 V>electrolysis potential > +0.738 V

Additionally, it needs to be fully compatible with the used electrolyte to avoid any possibility of chemical degradation during the separation process. Several classes of electrolytes of organic or aqueous origin can be used. The aqueous electrolytes show higher radiation stability during the course of the electrolysis compared to organic liquids, which results in fewer chemical impurities associated with the produced ^99m^Tc radioactive solution. However, their use is usually associated with the evolution of hydrogen gas, which lowers the pH of the electrolyte medium and may decrease the separation performance. For the working electrodes, they need to be chemically inert with considerable electrochemical and radiation resistance. The temperature of the medium should be adjusted sufficiently below the electrolyte boiling point to avoid its rapid evaporation, which results in unacceptable ^99m^Tc solution purity. The reaction time should be established to achieve the highest possible ^99m^Tc yield in the shortest period to minimize ^99m^Tc radioactivity loss (Chakravarty et al., 2012b; 2012c, 2011; 2010a; 2010b).

Based on this method, Chakravarty et al., 2010a, reported the production of ^99m^Tc from a ^99^Mo/^99m^Tc generator. The daughter extraction was performed in an electrochemical cell by applying a constant potential of 5 V in sodium hydroxide electrolyte (pH ∼ 13). The electrolysis process lasted for approximately 1 hour. Hence, the ^99m^Tc selectively accumulated on the platinum electrode (cathode). The ^99m^Tc deposited was recovered in a 0.9% saline solution. Then, it was purified with the use of an alumina column (Chakravarty et al., 2010a).

The prospect of ^99^Mo/^99m^Tc electrochemical-based generators provide an adequate ^99m^Tc yield in a non-carrier added form with a satisfactory purity level for radiopharmaceutical applications. In addition, their production capacity neither relies on the amount of the used sorbent, nor the extractant volume. Nevertheless, this production strategy is implicated with some opposing weaknesses that preclude its practical medical use. For example, the electrolysis step is accompanied by the evolution of oxygen and hydrogen gases, which may pose an explosion hazard. In addition, the oxygen gas may inhibit the reduction of ^99m^TcO_4_^-^ species, which drastically decreases the separation yield. Moreover, the working electrodes require tedious cleaning processes directly after each separation cycle. Furthermore, it is a relatively time-consuming production technique, including different consecutive separation and purification steps, which consequently result in a marked loss of ^99m^Tc radioactivity. Finally, the procedure needs high shielding requirements to reduce the exposure dose to the working personnel. For these reasons, this strategy strongly requires a very rigorous operating regime with a high degree of safety and precautionary measures and well-trained operators with high technical awareness of electro- and radiochemistry fundamentals.

##### 3.2.3.4 Supported Liquid Membrane generators

This generator system can be introduced as a modification or an updated version of the solvent extraction method (Dhami et al., 2007; Dutta and Mohapatra, 2013; Chang, 2016; Davarkhah et al., 2018). It involves the separation of ^99m^Tc species from ^99^Mo with the use of a membrane as a semi-stationary phase and two other mobile phases that are located on both sides. These three compartments can be described as follows (Sastre et al., 1998; Chaudry, 2000; Yassine, 2000):

1. The primary aqueous solution (the feeding phase): It consists of the ^99^Mo/^99m^Tc mixture.
2. The stationary porous membrane: This membrane is of hydrophobic nature and serves as a barrier between the other two liquids. It is incorporated with an organic liquid of a high selective tendency for ^99m^Tc and delivers the ^99m^TcO_4_^-^ ions from the feed solution to the receiving liquid. Meanwhile, it restricts the flow of ^99^Mo species.
3. The eluate receiving solution (the stripping phase): this partition contains the recovered ^99m^Tc.

The extraction process is performed in three simultaneous steps. In the first step, the ^99m^Tc species are selectively collected from the feed mixture by the extractant. Then, they travel through the membrane to the other side, where they are eventually exchanged with another species from the clean receiving solution. This journey of ^99m^Tc from the primary solution to the membrane and from the membrane to the receiving solution is only controlled by separation reaction kinetics without the help of any external driving force. Therefore, it is only attributed to the concentration difference on both sides of the membrane.

Many different supported liquid membrane frameworks have been studied to separate ^99m^Tc on the laboratory scale (Sastre et al., 1998; Gyves and Rodríguez, 1999; Chaudry, 2000; Yassine, 2000; Chen et al., 2001). Nonetheless, a number of inherent practical difficulties still ask for more research efforts. For instance, it is a time-consuming strategy and involves the loss of the majority of ^99m^Tc radioactivity attributable to the remarkably slow extraction kinetics. In addition, it supplies unsatisfactory ^99m^Tc separation yield with high ^99^Mo breakthrough and organic chemical impurities due to the low radiation resistance of the organic liquids and limited life-course of the used membrane. Furthermore, it generates substantial amounts of radioactive waste.

##### 3.2.3.5 Column chromatographic generators

Several ^99^Mo/^99m^Tc generator production strategies have been explored and developed over the last few decades. Nevertheless, the column chromatography-based generator type has attracted considerable interest as a reliable source to supply ^99m^Tc ready for nuclear medicine investigations. As a result, the chromatographic column generator is considered the most predominant choice for the hospitals and nuclear medicine departments owing to many valuable benefits, including (Nawar and Türler, 2019):

• One-step process: The ^99m^Tc radioactivity can be extracted in only one step, effectively minimizing the radioactive decay losses.
• Simple to use: The operation is feasible, user-friendly, and does not involve any operational difficulties or chemical and radiation hazards.
• Safe to use: The small generator size facilitates its shielding. Thereby, it can be handled and eluted safely with a minimum radiation dose to the operators.
• Rapid elution process: The generator offers a short elution time due to the fast separation kinetics of the daughter.
• High elution efficiency: The generator system provides a high separation yield of ^99m^Tc from its parent mixture with a favorable carrier-free grade.
• High eluate purity: The ^99m^Tc solution can be collected free from ^99^Mo with high chemical, radiochemical, and radionuclidic purity (without any need for post-elution purification steps).
• Small elution volume: The generator is designed to give the entire ^99m^Tc radioactivity yield in a very minute quantity with a high radioactive concentration adequate for immediate use, which avoids any further concentration challenges.
• Reproducible ^99m^Tc supply: The generator is capable of frequent multiple elutions with sustainable and stable supply in terms of ^99m^Tc yield and purity over its lifetime.
• Marginal generation of radioactive waste: It is reflected in a clean working environment and the absence of any contamination risk.

In generic terms, this generator system consists of two phases, i.e., a stationary phase (the sorbent material) and an external liquid phase (the eluent solution). The central concept is built on the retention of ^99^Mo radionuclide on a column incorporated with proper sorbent material. Then, the ^99^Mo decays to yield ^99m^Tc, followed by the elution of the generated daughter with a suitable isotonic saline solution. The separation of the ^99m^Tc species is mainly based on the difference between their low retention affinity on the column material and their high solubility in the eluent solution. The parent ^99^Mo exists in the molybdate form, M99oO42− , which in a weakly acidic medium, polymerizes to polymoybdate species, as follows (Dash and Chakravarty, 2016; Nawar and Türler, 2019):

7 M99oO42−+8 H+ ⇌Mo799O246−+4 H2O (23)

The formed species with six negative charges offer favorable conditions for interacting with the positively charged sorbent surface. After that, the polymoybdate Mo799O246− decays to form the technetium pertechnetate (^99m^TcO_4_^-^), which possesses only one negative charge. Hence, the binding ability of ^99m^TcO_4_^-^ becomes weaker than ^99^Mo_7_O_24_^6-^ with the stationary phase (Dash and Chakravarty, 2016). Then, an isotonic saline solution is subsequently passed through the column to replace the weakly bound ^99m^TcO_4_^-^ species with Cl^−^ ions to produce sodium pertechnetate (Na^99m^TcO_4_), leaving the ^99^Mo on the column, according to the equations:

Mo799O246−+6 R+ →R6Mo799O24 →β−  RT99mcO4−+5 R+ (24)

RT99mcO4−+NaCl →NaT99mcO4+RCl (25)

The generator performance greatly depends on the sorption–elution kinetics and the degree of the sorbent selectivity for the parent Mo (containing ^99^Mo). Therefore, the careful selection of the column material is the prime factor for the success of this generator system. Favorable sorbent material should have a considerable selectivity for Mo and, at the same time, a very limited or no selective capability for ^99m^Tc. Based on this parameter, an optimum separation environment can be established between the two radionuclides, and thereby, the ^99m^Tc can be extracted in high purity. Furthermore, fast elution kinetics of the daughter can be obtained.

Moreover, the sorbent material should show a high sorption capacity for Mo (containing ^99^Mo). The sorption capacity indicates the quantity of Mo the column material can retain. It mainly depends on the number of available active sites on the sorbent surface for the parent element. Higher sorption capacity is directly reflected by a lesser amount of column material needed to achieve the desirable radioactivity level. Consequently, it is translated to a higher radioactive concentration of the eluted ^99m^Tc solution.

Furthermore, the column material needs to have a high radiation resistance and suitable chemical stability to withstand the intense radioactive irradiation and the chemical environments (Nawar and Türler, 2019). In this regard, varieties of column chromatographic generators have been developed. The differences among these generators are the sorbent material and the origin of ^99^Mo. The following subsections highlight different column chromatographic generators approaches and their utilization to produce ^99m^Tc for medical use.

###### 3.2.3.5.1 Alumina-based chromatographic generators

This strategy is the dominant option to afford a reliable and widespread supply of ^99m^Tc for medical harnessing. The generator system consists of a small column filled with a few grams of acidic alumina (Al_2_O_3_) as a sorbent matrix. First, the parent ^99^Mo is extracted from the fission mixture in an alkaline solution as a molybdate anion (^99^MoO_4_^2−^). Then, this solution is acidified to maintain favorable sorption conditions. After that, it is loaded on the pre-acidified column, and finally, the ^99m^Tc can be easily eluted in the form of (^99m^TcO_4_^−^) using 0.9% NaCl solution (IAEA, 2013b; Nawar and Türler, 2019).

The earliest produced generator was first eluted with 1 M HNO_3_. Then, the eluent was changed to HCl until Hammersmith Hospital-London reported the efficient use of the isotonic NaCl solution. These attempts were designed to provide a perfectly compatible eluate solution with the human biological system and to lower the contamination impurity level of ^99^Mo in the eluted ^99m^Tc solution (Matthews and Mallard, 1965; Richards et al., 1982).

**FIGURE 5:**
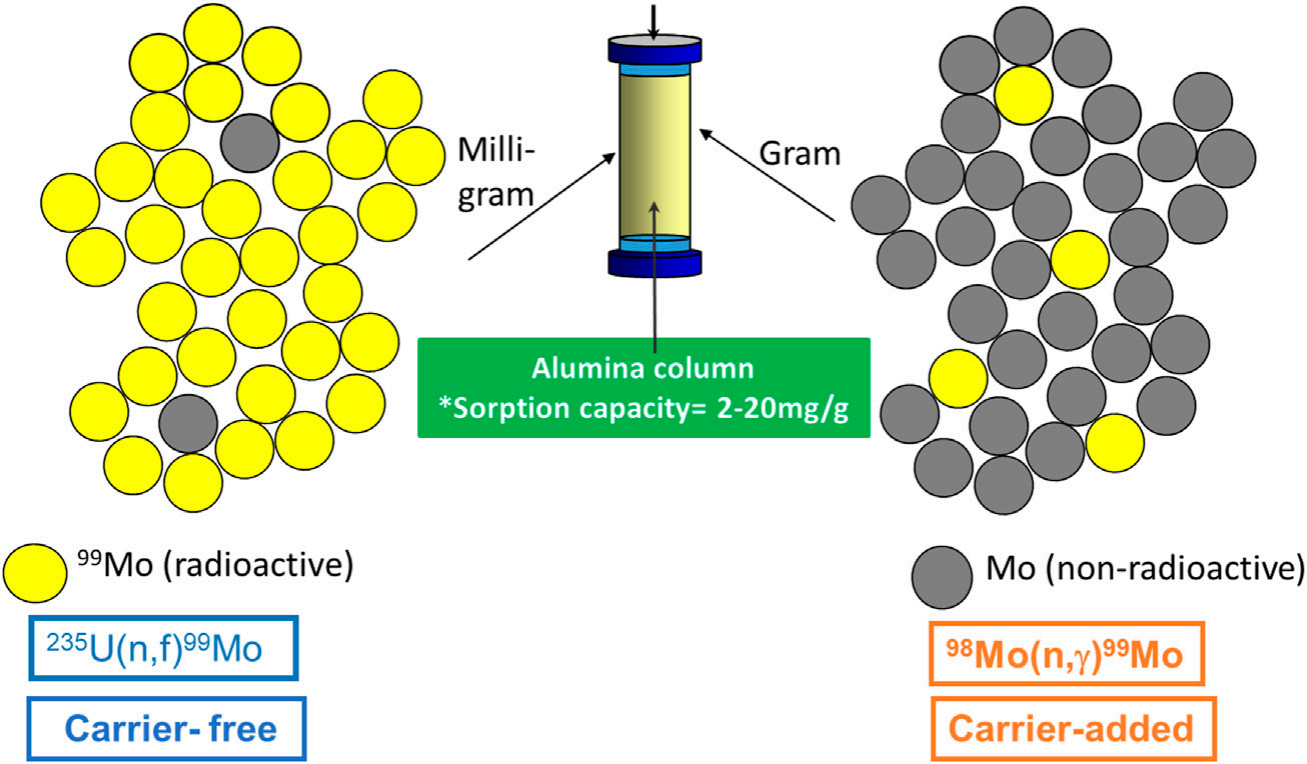
Comparison between the alumina generators based on neutron-capture-produced and fission-produced ^99^Mo.

As previously mentioned in Section 2.1.1, the fission production method is associated with a variety of critical difficulties. In addition, it is only restricted to very few centers worldwide, and it is not formally adopted as an integrated routine production approach in developing countries. In order to overcome these challenges, alternative strategies have been proposed using neutron-capture-produced ^99^Mo of low and medium-specific activity. Figure 5 graphically illustrates a comparison between the alumina generators based on neutron-capture-produced and fission-produced ^99^Mo.

The first ^99m^Tc generator, which was based on the loading with neutron-capture-produced ^99^Mo onto the conventional alumina columns, was produced and commercially distributed by Nuclear Consultants Inc. of St Louis, which was merged afterward with Mallinckrodt and Union Carbide Nuclear Corporation in New York in 1966 (Richards et al., 1982). Owing to the distinctly limited sorption capacity of conventional alumina (2–20 mg Mo/g) (Molinsky, 1982) and the fairly low specific activity of ^99^Mo, the effective practical implementation of this technology suffered from a variety of inherent roadblocks. These limitations include the vital need for considerable amounts of alumina to build a useful generator of a suitable radioactivity level, which results in the elution of the ^99m^Tc in a relatively large volume with a very low Radioactive Concentration (RAC), which accompanies by more tedious post elution concentration steps. However, the use of enriched ^98^Mo targets increase the radioactivity of the produced ^99^Mo and accordingly improves the elution profile of the eluted ^99m^Tc, the enriched ^98^Mo target are costly and cannot be easily recycled (Maoliang, 1997; NAP, 2018). Therefore, these ^99m^Tc generators, which were built on traditional alumina column and neutron-capture-produced ^99^Mo, were abandoned.

###### 3.2.3.5.2 Gel-type chromatographic generators

The possibility of using neutron-capture-produced ^99^Mo to produce chromatographic ^99m^Tc generators has promoted the need to explore the gel generator concept (Evans et al., 1987; Moore et al., 1987; Boyd, 1997; Maoliang, 1997). Generally, the gel generator strategy is a multi-step production approach based on using ^99^Mo-labeled molybdate matrices as column materials. The preparation of these matrices involves many consecutive steps, such as the dissolution of the irradiated ^99^Mo targets and the precipitation of insoluble solid material with the help of chemical reagents, followed by several filtrations, drying, fragmentation, and column packing steps. At this point, the column is ready for ^99m^Tc elution (IAEA, 1999).

In more detail, the ^99^Mo-molybdate matrices can be prepared *via* two main approaches:

• Pre-irradiation formed gel technology: This method includes the preparation of an inactive molybdate gel matrix. After that, it is irradiated in the nuclear reactor and consecutively packed into a column for ^99m^Tc extraction (Narasimhan et al., 1984; Vanaja et al., 1987; Shafiq and Yousif, 1995).
• Post-irradiation formed gel technology: This route starts with the irradiation of the ^98^Mo target. Then, the molybdate-^99^Mo matrix is precipitated from its solution as an insoluble gel material. After that, the dried precipitate is packed into a column for ^99m^Tc elution (Boyd, 1982a, 1982b, 1987, 1997; Evans et al., 1987; Moore et al., 1987; El-Kolaly, 1993; IAEA, 1995; Junior et al., 1998; Monroy-Guzman et al., 2003, 2007, 2008; Saraswathy et al., 2004; Davarpanah et al., 2009; Mostafa et al., 2016).

These technologies introduce some critical limitations. For example, the former method suffers from the degradation of the pre-formed gel. The intense radiation level in the reactor induces the reduction of Mo(VI) and Tc(VII) to lower oxidation states, which inevitably results in unsatisfactory elution performance and extremely low ^99m^Tc elution yield owing to the retention of the ^99m^Tc on the column matrix. Moreover, the irradiation of the entire material strongly leads to the production of undesirable radioactive impurities, which consequently lower the radionuclidic purity of the eluted ^99m^Tc solution. The post-irradiation formed gel technology is a multi-step procedure that is hampered by the loss of ^99^Mo radioactivity, generation of significant amounts of radioactive waste, and excessive radiation exposure to the working personnel (Boyd, 1987; Evans et al., 1987; Zaidi et al., 1990; Saraswathy et al., 2004; Davarpanah et al., 2009). In conclusion, the perceived need for high purity and an adequate radioactive concentration of the produced ^99m^Tc from the generator set severe technical limitations on the mandatory minimum of the required specific activity of ^99^Mo. As a result, this strategy could not have been able to afford a reliable supply of ^99m^Tc for clinical use.

###### 3.2.3.5.3 Generator systems using nanomaterial-based sorbents

Because of the fact that the sorbent material is the heart of column chromatographic generators, many recent studies have focused on developing a new generation of sorbents that possess a high ^99^Mo sorption capacity to facilitate the efficient utilization of ^99^Mo of low and medium-specific activity. These materials are fabricated on the basis of the nanotechnology. Nano-materials attracted considerable attention due to their unique characteristics and innovative applications in multi-disciplinary fields, such as the drug industry, water decontamination development, and different materials production technology (Kharisov et al., 2014). Currently, more than 1000 nanotechnology-based products have become commercially available (Sebastian et al., 2014).

Nanosorbents are size-dependent materials, and their properties not only depend on their chemical composition, but also on their nano-size range and shape. As the particle size decreases, the relative surface area increases, which results in a more significant number of active atoms present on the material surface. These active sites can be translated into a considerable chemical reactivity. In other words, since the chemical reactions occur at the surface, a certain quantity of nano-sized sorbents will be much more reactive than the same amount of macro or micro-scaled materials (Bhatia, 2016).

Based on the formerly mentioned strategies for preparing column chromatographic generators, it is pertinent to point out that the dominant practical challenge is the relatively low sorption capacity of the traditionally used sorbents. This low capacity requires high specific activity ^99^Mo to produce ^99m^Tc generators of a suitable radioactivity level. This ^99^Mo can only be made available from the fission of ^235^U. Some other technologies attempted using neutron-capture-produced ^99^Mo. However, their applicability is associated with many potential limitations, as discussed above. These problems can be avoided with the use of nanosorbents. That is because reducing the particle size to the nano-scale range offers new opportunities and gives this group of sorbents a number of unique characteristics. For example, these sorbents exhibit novel physical and chemical properties, resulting in extraordinary sorption reactivity and rapid extraction kinetics. In addition, their high chemical and radiation stability draw a clear distinction between them and the conventional bulk material (Baron et al., 2008; Yousefi et al., 2012; Kharisov et al., 2014; Schmid, 2011). Therefore, the use of nanoparticle-based sorbents can offer the following advantages:

• High parent sorption capacity and selectivity due to their large surface area and the increased number of active sites. Consequently, the preparation of a clinical-scale ^99^Mo/^99m^Tc generator using neutron-capture-produced ^99^Mo without the limitations of the sorbent capacity can possibly be achieved.
• Simple ^99^Mo loading process with a minor generation of radioactive waste.
• Considerable chemical stability under different concentrations of acidic and basic solutions leads to the production of ^99m^Tc with high chemical purity.
• High radiation resistance prevents ^99^Mo breakthrough and provides the eluted ^99m^Tc with adequate radionuclidic purity for nuclear medicine applications.
• Favorable elution performance with a sharp ^99m^Tc elution profile, which supplies the ^99m^Tc eluate with a high radioactive concentration and limits any need for additional concentration efforts.

Table 9 underlines several research attempts using different nanomaterial-based sorbents to develop ^99^Mo/^99m^Tc generator systems (Chakravarty et al., 2008, 2010c, 2012a, 2013; Fasih et al., 2016; Nawar, 2021). It can be observed that the new sorbents exhibit a much higher sorption capacity for ^99^Mo compared to the commercially used alumina. Its sorption capacity ranges from (2–20 mg Mo/g) (Molinsky, 1982). Furthermore, the eluted ^99m^Tc can be collected with adequate elution yield, radionuclidic, radiochemical, and chemical purity levels.

**TABLE 9:** The summary of ^99^Mo/^99m^Tc generators developed using nanosorbents: preparation, structural characterization, loading capacity, and elution quality control data.

Nano-sorbents	Method of preparation	Structural characteristics			Sorption capacity, (mg Mo/g)		Activity of ^99^Mo loaded, GBq (mCi)	Specific activity of ^99^Mo used, GBq/g of Mo	^99m^Tc Elution performance				Reference
									Elution yield, (%)	Level of ^99^Mo impurity in ^99m^Tc, (%)	Radio-chemical purity of ^99m^TcO_4_^-^, (%)	Level of chemical impurity in ^99m^Tc, (μg/ml)	
		Crystalline size, (nm)	Pore size, (nm)	Surface area, (m^2^.g^−1^)	Static	Dynamic							
CeO_2_	Controlled hydrolysis of Ce(NO_3_)_3_.6H_2_O under alkaline conditions	3.54	2.75	187	192 ± 10	84 ± 6	1.0 (27)	∼5	76.46 ± 1.1	<10^–3^	>99	Ce < 0.01	Nawar, (2021)
TiO_2_	Hydrolysis of TiCl_4_ in isopropyl alcohol medium	∼40	0.005	320	141 ± 2	78 ± 2	0.74 (20)	∼3	>84	Not detected	≥97.3	Ti < 0.01	Fasih et al. (2016)
Meso- Al_2_O_3_	Hydrolysis of Al(C_3_H_8_O)_3_ in presence of glucose template, followed by calcination	2 ± 1	3 ± 1	230 ± 10	225 ± 20	168 ± 12	26 (700)	18.5	>80	<10^–3^	>99	Al < 0.1	Chakravarty et al. (2013)
γ-Al_2_O_3_	Mechanochemical reaction of aluminium nitrate with ammonium carbonate	5 ± 1	0.4 ± 0.2	250 ± 10	205	150	13.0 (350)	11.1	>82	<10^–3^	>99	Al < 0.1	Chakravarty et al. (2012a)
t- ZrO_2_	Controlled hydrolysis of ZrOCl_2_.8H_2_O under alkaline conditions	8 ± 2	0.4 ± 0.1	340 ± 20	250 ± 10	80	9.25 (250)	17.8	>85	<10^–4^	>99	Zr < 0.1	Chakravarty et al. (2010c)
Tip	Controlled hydrolysis of TiCl_4_ in isopropyl alcohol medium	5 ± 1	0.4 ± 0.1	30 ± 2	110	75	1.1 (30)	∼6–13	75–80	<10^–3^	96.5–99.8	Ti < 0.1	Chakravarty et al. (2008)

Even though some milestones have been achieved, it is of importance to recognize that this approach is still in its infancy, and further research areas need to be covered. The developed methods are not currently standard practice in ^99^Mo/^99m^Tc generators. The generators capacities demonstrated in Table 9 are not sufficient for clinical use. Future studies should intend to scale up for clinical use, which typically involves ^99^Mo activities of 40–700 GBq. These studies need to investigate the performance of the column matrices in terms of ^99m^Tc purity and carefully assess its labeling efficacy using the conventional kit formulations. Since utilizing nano-sorbent materials and LSA ^99^Mo represents a new method to produce ^99m^Tc generators, local and international regulatory approvals are needed. This approval is a prerequisite before any clinical administration to the patients to prove that the quality and the efficacy of the produced ^99m^Tc are equal or even show some advantages over the ^99m^Tc obtained from the classical alumina column chromatographic generator containing fission-produced ^99^Mo (i.e., purity and dose cost).

### 3.3 Modified ^99^Mo/^99m^Tc separation systems

#### 3.3.1 ^99m^Tc master milker

**FIGURE 6:**
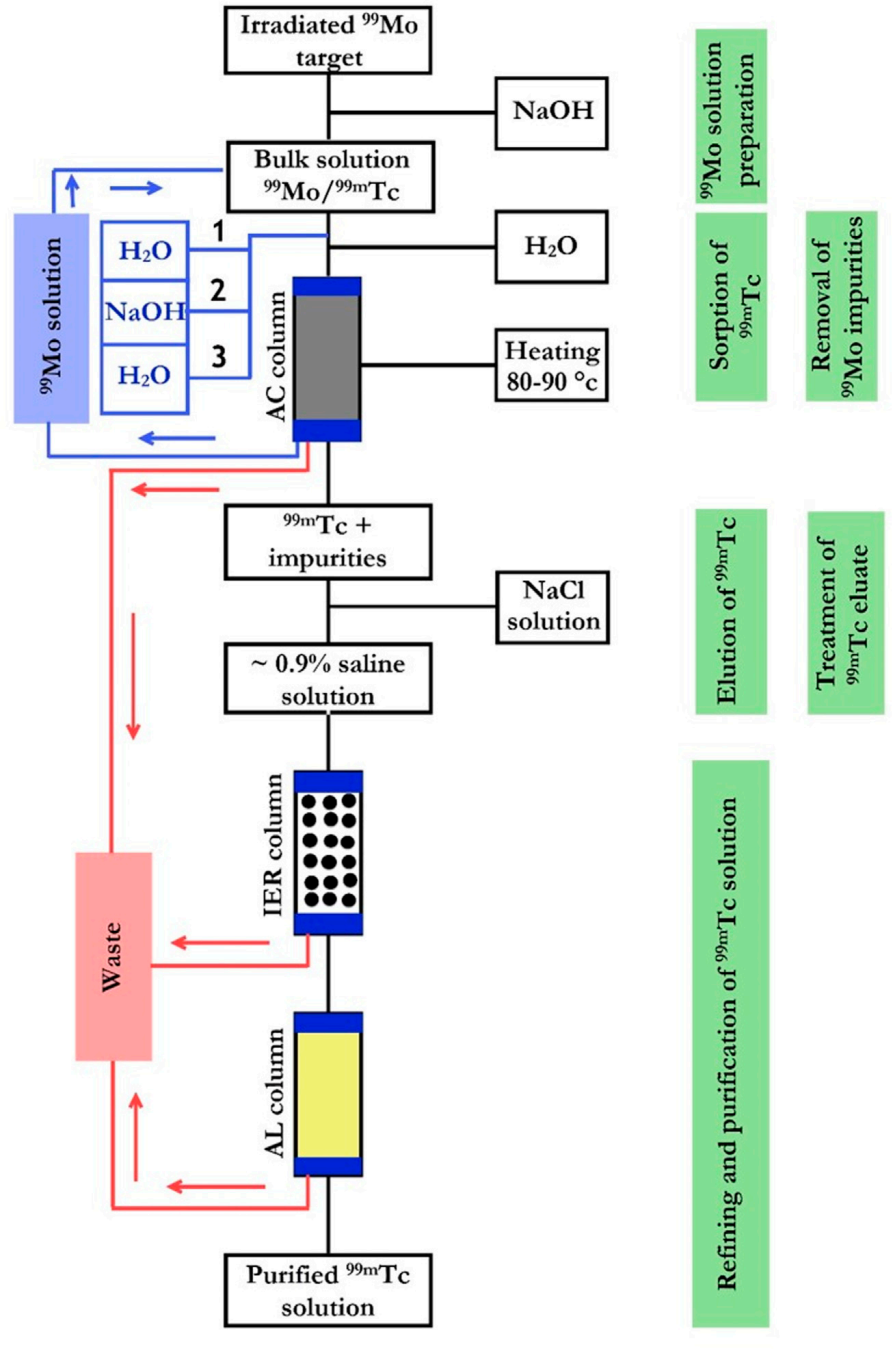
The schematic illustration of the TcMM process.

Many recent studies have focused on improving the ^99^Mo/^99m^Tc generator performance to provide an efficient extraction of ^99m^Tc. In this context, a modified version of a column chromatographic ^99m^Tc generator, namely the ^99m^Tc Master Milker (TcMM), was designed by Kaken Inc. Mito; Japan in 2014 (Tatenuma et al., 2016a, 2014). The TcMM technique is a new separation concept, which can provide a local supply of ^99m^Tc solution recovered from ^99^Mo of low and medium-specific activity. The idea is mainly built on the separation of ^99m^Tc from its parent through the use of three different column materials; Activated Carbon (AC), Ion Exchange Resin (IER), and activated acidic Alumina (AL) columns. Figure 6 illustrates the TcMM process scheme.

In more detail, TcMM is a multi-step process that involves the separation of ^99m^Tc from ^99^Mo in six primary stages, as follows (Sekimoto et al., 2017; Tatenuma et al., 2016b, 2014):

##### 3.3.1.1 Preparation of the ^99^Mo solution

Following the delivery of the irradiated ^99^Mo targets, a radioactive ^99^Mo/^99m^Tc mixture is prepared by dissolving the irradiated targets in 2M NaOH according to the following reaction:

M99oO3 +2NaOH  → Na2M99oO4 + H2O (26)

##### 3.3.1.2 Sorption of ^99m^Tc

The formed Na_2_^99^MoO_4_ solution is allowed to pass through a stainless steel column loaded with 5 g of AC, by which a particularly selective extraction of ^99m^Tc can take place. The AC column has high sorption efficiency towards ^99m^Tc compared to ^99^Mo and other radio-contaminants present in the solution. The reason for this selective sorption behavior capability is still under investigation.

##### 3.3.1.3 Removal of ^99^Mo impurities

During the second step, small amounts of ^99^Mo contaminants may be adsorbed on the activated charcoal column. In order to remove these impurities, three successive elution steps have to be performed. In the first step, the column is rinsed with a sufficient amount of water. Then, ^99^Mo is eluted with sodium hydroxide solution. Finally, the column is rewashed with water to remove any residual quantities of ^99^Mo and NaOH. The flushed ^99^Mo solution can be allowed to decay to be reused again. This step may be repeated many times until the ^99^Mo no longer has enough activity to be utilized.

##### 3.3.1.4 Elution of ^99m^Tc

In this step, the stainless steel column containing the activated carbon is heated up to 80–90°C. In the meantime, a sufficient amount of water is passed through the column. Hence, the ^99m^Tc radioactivity can be collected as a warm, weakly alkaline solution.

##### 3.3.1.5 Treatment of the ^99m^Tc eluate

The obtained solution from the previous step is mixed with sodium chloride solution to form a 0.9% saline solution.

##### 3.3.1.6 Refining and purification of ^99m^Tc

After completing the elution process (step 4), the ^99m^Tc solution may still contain some undesirable radio-impurities such as ^92m^Nb, ^95^Nb, and ^96^Nb, which arise from the neutron activation of stable radioisotopes of molybdenum, in addition to the parent ^99^Mo. In order to purify the ^99m^Tc eluate from these radio-contaminants, additional purification steps have to be carried out. Therefore, the resulting ^99m^Tc solution from step 5 is allowed to pass through two successive columns; IER and AL columns, respectively.

The TcMM technology offers a new possibility for separating highly purified ^99m^Tc from ^99^Mo of low and medium-specific activity. The produced ^99m^Tc shows high labeling efficacy with several pharmaceutical kits. Nevertheless, this technology still requires approval for making human-use ^99m^Tc radiopharmaceuticals. The TcMM can utilize ^99^Mo radioactivity in the range of kBq to TBq and produce ^99m^Tc of a maximum of 500 Ci (18.5 TBq) per batch (Tatenuma et al., 2016a, 2014). However, the produced ^99m^Tc can only be distributed locally to the usage sites near the separation centers. In addition, the loss of some of the ^99m^Tc radioactivity during the multi-separation and purification steps and the transportation to more distant usage sites is a marked disadvantage.

#### 3.3.2 Northstar radiogenix

Another new modified version of the ^99m^Tc generator is the Radiogenix system, which was designed as a closed system by Northstar Medical Isotopes, LLC, Beloit, Wisconsin; United States (Harvey, 2019). Northstar has received approval from the US Food and Drug Administration (FDA) for using the Radiogenix system to supply ^99m^Tc for nuclear medicine applications (Harvey, 2019; Karcz, 2019). Northstar Radiogenix is an automated, computer-controlled platform designed to separate ^99m^Tc daughter from non-uranium produced ^99^Mo of low specific activity in a highly alkaline solution.

Radiogenix is based on separating ^99m^Tc with the use of two different separation cartridges, namely the Primary Separation Cartridge (PSC) and ^99m^Tc Product Cartridge (TPC) (Northstar, 2020). Once the irradiated Mo targets are received, they are dissolved, and the ^99^Mo solution is contained inside the source vessel. After that, the ^99^Mo/^99m^Tc mixture is pumped through PSC containing Polyethylene Glycol (PEG)-based Aqueous Biphasic Extraction Chromatography (ABEC) resin. This resin selectively retains the ^99m^Tc, and the parent, ^99^Mo, passes through into the transfer vessel (Rogers et al., 1997; Morley et al., 2012; ANL, 2016). Then, with a stripping solution, ^99m^Tc is washed off from the ABEC resin and passed through the TPC into the ^99m^Tc collection vial. This step ensures the purification of ^99m^Tc from any ^99^Mo impurities. Eventually, the ^99^Mo is returned to the source vessel for reuse (Northstar, 2020). When the ^99^Mo solution reaches its expiration date and is no longer usable, it is returned to the manufacturer, primarily when enriched Mo targets are used (ANL, 2020).

Northstar Radiogenix is a high-tech technology that provides high-quality ^99m^Tc concentrations similar to the high-specific activity-based generators. Nevertheless, it has a physically large design. Moreover, since this method involves a different system and new protocol, the main question is whether the nuclear medicine community will be willing to adopt this approach in the near future.

## 4 Summary, conclusions, and future perspectives

This paper highlights the fundamental role of ^99m^Tc in medical investigations and its potential production strategies. ^99m^Tc (T_1/2_ = 6.01 h) is the most widely used diagnostic radionuclide in nuclear medicine. The ^99m^Tc labeled radiopharmaceuticals are prepared mostly in-house by using kit formulations. ^99m^Tc is conveniently derived from ^99^Mo/^99m^Tc generator systems. The parent ^99^Mo (T_1/2_ = 65.94 h) is obtained by chemical separation from neutron-irradiated ^235^U targets, by which Gram quantities of highly enriched ^235^U targets are irradiated in the thermal spectrum of research reactors. Then, it is shipped to special processing facilities, where the ^99^Mo is chemically separated and purified. The obtained specific activity of ^99^Mo is very high. Nonetheless, the process is associated with generating substantial amounts of nuclear waste, and the handling of ^235^U generates serious proliferation issues. Furthermore, the global ^99^Mo supply is fragile and insecure, as it only relies on few aging nuclear reactors. Since the ^99m^Tc supply depends significantly on the fission-produced ^99^Mo availability, it seriously suffered during renewed emergency periods, such as the past ^99^Mo production crisis and the current COVID-19 pandemic. To solve this challenge, many alternative ideas were put forward to mediate the problem. However, the commercial aspect should not be overlooked. These new methods to supply ^99m^Tc should be competitive in terms of production volume, price, and ease of applicability for the end-user. One method is to obtain ^99m^Tc by direct cyclotron production via the ^100^Mo (p,2n)^99m^Tc reaction. However, many nuclear medicine departments are hard to persuade to switch from the column ^99m^Tc generators and kit formulations to the ^99m^Tc-radiopharmaceuticals produced in centralized radiopharmacies that obtain ^99m^Tc by this route. Mainly, its supply advantage is limited to the centers close to the production site, and the daily transport costs can develop into a significant cost factor. In addition, this strategy still seeks to design a realistic supply plan that can deal with the production difficulties and find innovative logistic solutions to meet rapid product delivery requirements. Thereby, this strategy is not clearly the answer for a routine ^99m^Tc supply.

In order to establish a sufficient ^99m^Tc supply sustainably, two challenges need to be optimized:

• First, enhance the ^99^Mo economy by guaranteeing a reliable and decentralized worldwide supply source of Low Specific Activity (LSA) ^99^Mo. This concept can be made available by fostering the neutron irradiation of ^98^Mo targets in nuclear power reactors. Commercial nuclear power reactors provide intense neutron fluxes. More importantly, high fluxes of epi-thermal neutrons are available. Due to the activation of ^98^Mo solely, no chemical separation is required, and consequently, very marginal quantities of radioactive waste are generated. In addition, this method is proliferation-resistant, as no fissile materials are involved. Moreover, it needs less technical arrangements and financial budgets compared to the uranium fission approach. Another promising approach aims at producing ^99^Mo through photonuclear reactions through the ^100^Mo(γ,n)^99^Mo reaction. The currently available specific activity is about 6 Ci/g of ^99^Mo after a 24 h bombardment. The ^99^Mo produced has a low specific activity. Therefore, the issue of the low sorption capacity of the conventional sorbents persists (2–20 mg Mo/g).
• Second, the key lies in developing a new generation of column materials that possess high sorption capacity and selectivity to recompense for the low specific activity of the obtained ^99^Mo. A promising approach is the development of a chromatographic column ^99m^Tc generator with the use of nanomaterial-based sorbents. The use of nanomaterial-based sorbents deserves considerable attention due to their high sorption capacity, which arises from their large surface area, high radiation resistance, and chemical stability compared to conventionally used sorbents. These unique characteristics could be the cornerstone towards developing a new generation of ^99^Mo/^99m^Tc generators that produce ^99m^Tc with high purity and radioactivity concentration adequate for existing kit formulations. Consequently, this approach would encourage the possibility of using ^99^Mo of low specific activity without the limitations of the sorbent capacity.

Since the ^99m^Tc production techniques are usually selected based on different economic, technical, and logistical circumstances, the proposed solution in this article is inexpensive, realistic, and could be implemented in a short period. Furthermore, it has the potential to ensure a secure supply source of clinical-grade ^99m^Tc for millions of patients worldwide, especially for many countries which do not have access to fission ^99^Mo technology.
